# Lipocalin-2 is an essential component of the innate immune response to *Acinetobacter baumannii* infection

**DOI:** 10.1371/journal.ppat.1010809

**Published:** 2022-09-02

**Authors:** Jessica R. Sheldon, Lauren E. Himmel, Dillon E. Kunkle, Andrew J. Monteith, K. Nichole Maloney, Eric P. Skaar

**Affiliations:** 1 Department of Pathology, Microbiology, and Immunology, Vanderbilt University Medical Center, Nashville, Tennessee, United States of America; 2 Vanderbilt Institute for Infection, Immunology, and Inflammation, Vanderbilt University Medical Center, Nashville, Tennessee, United States of America; 3 Vanderbilt Institute of Chemical Biology, Vanderbilt University, Nashville, Tennessee, United States of America; Emory University School of Medicine, UNITED STATES

## Abstract

*Acinetobacter baumannii* is an opportunistic pathogen and an emerging global health threat. Within healthcare settings, major presentations of *A*. *baumannii* include bloodstream infections and ventilator-associated pneumonia. The increased prevalence of ventilated patients during the COVID-19 pandemic has led to a rise in secondary bacterial pneumonia caused by multidrug resistant (MDR) *A*. *baumannii*. Additionally, due to its MDR status and the lack of antimicrobial drugs in the development pipeline, the World Health Organization has designated carbapenem-resistant *A*. *baumannii* to be its priority critical pathogen for the development of novel therapeutics. To better inform the design of new treatment options, a comprehensive understanding of how the host contains *A*. *baumannii* infection is required. Here, we investigate the innate immune response to *A*. *baumannii* by assessing the impact of infection on host gene expression using NanoString technology. The transcriptional profile observed in the *A*. *baumannii* infected host is characteristic of Gram-negative bacteremia and reveals expression patterns consistent with the induction of nutritional immunity, a process by which the host exploits the availability of essential nutrient metals to curtail bacterial proliferation. The gene encoding for lipocalin-2 (*Lcn2*), a siderophore sequestering protein, was the most highly upregulated during *A*. *baumannii* bacteremia, of the targets assessed, and corresponds to robust LCN2 expression in tissues. *Lcn2*^*-/-*^ mice exhibited distinct organ-specific gene expression changes including increased transcription of genes involved in metal sequestration, such as *S100A8* and *S100A9*, suggesting a potential compensatory mechanism to perturbed metal homeostasis. *In vitro*, LCN2 inhibits the iron-dependent growth of *A*. *baumannii* and induces iron-regulated gene expression. To elucidate the role of LCN2 in infection, WT and *Lcn2*^-/-^ mice were infected with *A*. *baumannii* using both bacteremia and pneumonia models. LCN2 was not required to control bacterial growth during bacteremia but was protective against mortality. In contrast, during pneumonia *Lcn2*^*-/-*^ mice had increased bacterial burdens in all organs evaluated, suggesting that LCN2 plays an important role in inhibiting the survival and dissemination of *A*. *baumannii*. The control of *A*. *baumannii* infection by LCN2 is likely multifactorial, and our results suggest that impairment of iron acquisition by the pathogen is a contributing factor. Modulation of LCN2 expression or modifying the structure of LCN2 to expand upon its ability to sequester siderophores may thus represent feasible avenues for therapeutic development against this pathogen.

## Author summary

A lack of therapeutic options has prompted the World Health Organization to designate multidrug-resistant *Acinetobacter baumannii* as its priority critical pathogen for research into new treatment strategies. The mechanisms employed by *A*. *baumannii* to cause disease and the host tactics exercised to constrain infection are not fully understood. Here, we further characterize the innate immune response to *A*. *baumannii* infection. We identify nutritional immunity, a process where the availability of nutrient metals is exploited to restrain bacterial growth, as being induced during infection. The gene encoding for lipocalin-2 (*Lcn2*), a protein that can impede iron uptake by bacteria, is highly upregulated in infected mice, and corresponds to robust LCN2 detection in the tissues. We find that LCN2 is crucial to reducing mortality from *A*. *baumannii* bacteremia and inhibits dissemination of the pathogen during pneumonia. In wild-type and *Lcn2*-deficient mice, broader transcriptional profiling reveals expression patterns consistent with the known response to Gram-negative bacteremia. Although the role of LCN2 in infection is likely multifactorial, we find its antimicrobial effects are at least partly exerted by impairing iron acquisition by *A*. *baumannii*. Facets of nutritional immunity, such as LCN2, may be exploited as novel therapeutics in combating *A*. *baumannii* infection.

## Introduction

*Acinetobacter baumannii*, a Gram-negative opportunistic pathogen, was first recognized as a distinct species in 1986 [1]. Since that time, *A*. *baumannii* has transitioned from being regarded as a relatively rare nosocomial pathogen of limited concern, to being recognized as a serious global health threat. This cause for alarm has arisen due to an increase in severe community-acquired *A*. *baumannii* infections [2–4], and more concerningly through the rapid acquisition of multidrug resistance by the organism [5–8]. Consequently, the World Health Organization recently assigned carbapenem-resistant *A*. *baumannii* to the top of its list of bacteria urgently requiring research and development into novel therapeutic approaches, designating it a priority “critical” pathogen [9]. Similarly, in 2019 the Centers for Disease Control and Prevention increased its assessment of the threat posed by antibiotic resistance in *A*. *baumannii* from “serious” to its highest designation of “urgent” [10]. With a propensity to cause ventilator-associated pneumonia (VAP) and infiltrate intensive care units (ICUs), the immediacy of the risk posed by this pathogen has been thrust to the fore by several outbreaks of hospital-acquired carbapenem-resistant *A*. *baumannii* associated with the ongoing COVID-19 pandemic [11,12]. Further, both the prevalence of *A*. *baumannii* as the causative agent of VAP and its overall resistance to antibiotics have increased since 2019 [13–16]. Together these observations indicate that *A*. *baumannii* is a major player in the scourge of antimicrobial resistance (AMR) globally and highlights a need for research and development into new therapeutic strategies to counter infections by this pathogen.

Until recently, *A*. *baumannii* has garnered relatively little attention from the research community and thus the mechanisms used by the bacterium to facilitate pathogenesis are not well understood. To develop novel treatment strategies to combat this pathogen, a more comprehensive understanding of both how *A*. *baumannii* survives within the host, and how the host counters *A*. *baumannii* infection are required. Unlike professional pathogens, *A*. *baumannii* lacks traditional virulence factors, such as toxins. Instead, it is thought that *A*. *baumannii* adopts a strategy known as “persist and resist,” whereby pathogenesis of the organism is facilitated primarily by its ability to survive unfavorable conditions such as nutrient deprivation, desiccation, pH extremes, disinfection, and oxidative stress [17]. Indeed, our laboratory and others have determined that the acquisition of essential nutrient metals such as iron, zinc, and manganese contribute to the virulence of *A*. *baumannii in vivo* [18–26], whereas exposure to disinfectants such as ethanol has been linked to increased biofilm formation and growth of the pathogen both *in vitro* and *in vivo* [27–29]. The capacity of *A*. *baumannii* to withstand desiccation and disinfection likely creates a nosocomial reservoir [30–32], whereby surviving populations of bacteria may rapidly establish infection upon return to a more favorable growth environment within the host. This notion is supported by the high prevalence of surface contamination by *A*. *baumannii* in these environments, and by the observation that use of ICU beds previously occupied by *A*. *baumannii* infected patients is a major risk factor for subsequent patients developing *A*. *baumannii* VAP [33,34].

To establish an infection, invading microbes must either evade or overwhelm the host immune response. Although the innate immune response is crucial to the early defense against many respiratory pathogens [35], its role in controlling *A*. *baumannii* infections is incompletely understood. As with other ESKAPE pathogens (*Enterococcus faecium*, *Staphylococcus aureus*, *Klebsiella pneumoniae*, *A*. *baumannii*, *Pseudomonas aeruginosa* and *Enterobacter* spp.), *A*. *baumannii* predominantly infects immunocompromised and/or critically ill ICU patients [36–39]. As such, much of our understanding of the innate immune response to *A*. *baumannii* infection has been characterized through the use of mice or cell lines disrupted for distinct pathways, or using animals treated with immunosuppressive agents [reviewed in 40,41]. These studies have revealed that neutrophils are critical to host resistance to *A*. *baumannii*, where in both murine and porcine models, depletion of neutrophils through treatment with either cyclophosphamide or anti-neutrophil antibodies (anti-GR1 or anti-Ly6G) reduces the barrier to infection and exacerbates disease severity [42–46]. Indeed, neutrophils are rapidly recruited to the site of infection during *A*. *baumannii* pneumonia and depletion of these cells leads to a reduction in neutrophil-recruiting chemokines and cytokines [42,43,47,48]. The findings in animal models of infection recapitulate what is observed in human patients, where neutropenia is both a predisposing factor and a predictor of negative outcomes for *A*. *baumannii* infection, further highlighting the importance of these cells in host resistance [49,50].

Although it is evident that neutrophils play an important role in host immunity against *A*. *baumannii* infections, further research is required to help elucidate the bactericidal mechanisms employed to facilitate clearance. One potential function of neutrophils in their interaction with *A*. *baumannii* is to inhibit bacterial replication through a process known as nutritional immunity. Nutritional immunity is a facet of the innate immune response that involves exploiting the availability of essential metals to either starve foreign invaders of these growth-promoting nutrients or use their inherent reactivity to intoxicate the pathogen [51–53]. Neutrophils are a reservoir for key proteins that function in nutritional immunity including calprotectin, lactoferrin, and lipocalin-2 (LCN2; for a more comprehensive description of key players in nutritional immunity, see S1 Table). Calprotectin is a heterodimer of the subunits S100A8 and S100A9, comprises up to 60% of the soluble cytosolic fraction of neutrophils, and exerts its bacteriostatic activity through the sequestration of transition metals including zinc, manganese, and iron [54,55]. Through imaging mass spectrometry, S100A8 and S100A9 have both been observed in *A*. *baumannii* infected tissues and their abundance correlates with bacterial burdens [26,56]. Further, calprotectin contributes to metal-dependent growth inhibition of the pathogen *in vitro*, which in turn upregulates its metal starvation responses upon exposure to the protein complex [23–26,57–59]. Lactoferrin and LCN2 both function predominantly in iron sequestration, where lactoferrin is a glycoprotein that binds metals largely at mucosal surfaces but is also found as a component of neutrophil secondary granules and decorating neutrophil extracellular traps (NETs) [60,61]. *In vitro*, lactoferrin inhibits the growth of *A*. *baumannii* in an iron-dependent manner [19]. LCN2 (also known as siderocalin, neutrophil-associated gelatinase lipocalin (NGAL), mouse oncogene 24p3, and uterocalin) is expressed by neutrophils and is responsible for sequestering siderophores, small iron-chelating molecules that are produced and released by bacteria and other organisms, in an effort to fulfil their metabolic requirements and to maintain iron homeostasis [62–64]. The release of metal-sequestering proteins from neutrophils represents an important element of the antimicrobial activities of these cells in controlling *A*. *baumannii* infection.

Given the tendency of *A*. *baumannii* to infect patients with weakened immune systems, the development of therapeutics that have the potential to supplement or modulate the innate immune response represents an attractive avenue for the creation of new treatments. However, as detailed above, our understanding of the interaction between *A*. *baumannii* and the host remains incomplete. While the use of immunocompromised mice in the study of host-*A*. *baumannii* interactions may appropriately model a proportion of patients infected with *A*. *baumannii* and has been invaluable to shaping our current understanding of *A*. *baumannii* pathogenesis, it confounds a comprehensive understanding of the immune factors that may contribute to host resistance to infection.

In this study, we aimed to further characterize the host response to *A*. *baumannii* infection using immunocompetent mouse models of bacteremia and pneumonia. Through the use of NanoString technology, we observed that mice infected systemically with wild-type (WT) *A*. *baumannii* exhibit transcriptional changes consistent both with the response to Gram-negative bacteremia and the induction of host nutritional immunity. Of the genes associated with the latter, the most highly upregulated gene was that encoding for LCN2. Robust expression of LCN2 in infected tissues was confirmed through immunohistochemistry (IHC) and digital image analysis, where multiorgan and multicellular upregulation was observed. Gene expression changes in the *Lcn2*^*-/-*^ mice suggest possible perturbations to both metal homeostasis and the immune response in these animals. *In vitro*, LCN2 was found to inhibit the iron-dependent growth of *A*. *baumannii*, as well as induce iron-regulated gene expression, suggesting that it may help to counteract the robust iron scavenging capabilities of the pathogen. Although disruption of *Lcn2* expression did not impact the number of bacteria recovered from tissues of mice infected using a model of bacteremia, *Lcn2*^*-/-*^ mice were more susceptible to mortality from this infection. Conversely, *Lcn2*-deficient mice failed to control the survival and dissemination of *A*. *baumannii* during pneumonia, where higher bacterial burdens were recovered in the kidneys, heart, liver, spleen, lungs, and blood. Together these findings suggest that nutritional immunity, and specifically LCN2, play critical roles in the innate immune response to *A*. *baumannii* and that the influence of this neutrophil-associated protein on the outcome of infection is likely multifactorial.

## Results

### Gene expression changes in mice infected with *A*. *baumannii* are characteristic of Gram-negative bacteremia

To better characterize the innate immune response to *A*. *baumannii* infection, we employed a murine model of bacteremia to assess for changes in host gene expression during infection using NanoString technology. NanoString directly detects targeted transcripts using color-coded molecular signatures and digital detection without the need for amplification, allowing for gene expression analysis from complex biological samples [65,66]. We selected the nCounter Myeloid Innate Immunology panel [66], which provides comprehensive coverage of the innate immune response of myeloid-derived cells, targeting 754 murine transcripts and 19 different pathways or processes including, but not limited to, growth factor signaling, lymphocyte activation, antigen presentation, pathogen response, and complement activation [67]. In preparation for transcriptional profiling using NanoString, female C57BL/6J mice were either mock-infected with phosphate buffered saline (PBS) or infected with 2 x 10^8^–5 x 10^8^ colony forming units (CFU) of WT *A*. *baumannii* in PBS. Mice were infected by intravenous injection, using an established model of *A*. *baumannii* bacteremia [19] and approximating the common presentation of catheter-associated bloodstream infections by this pathogen [38]. The infection was allowed to proceed for 24 h, mice were humanely euthanized, and the kidneys, heart, and liver harvested. RNA was extracted from the tissues and hybridized to the Myeloid Innate Immunology CodeSet, followed by processing of the samples and analysis of the results as per the directions of the manufacturer [66].

**Fig 1:**
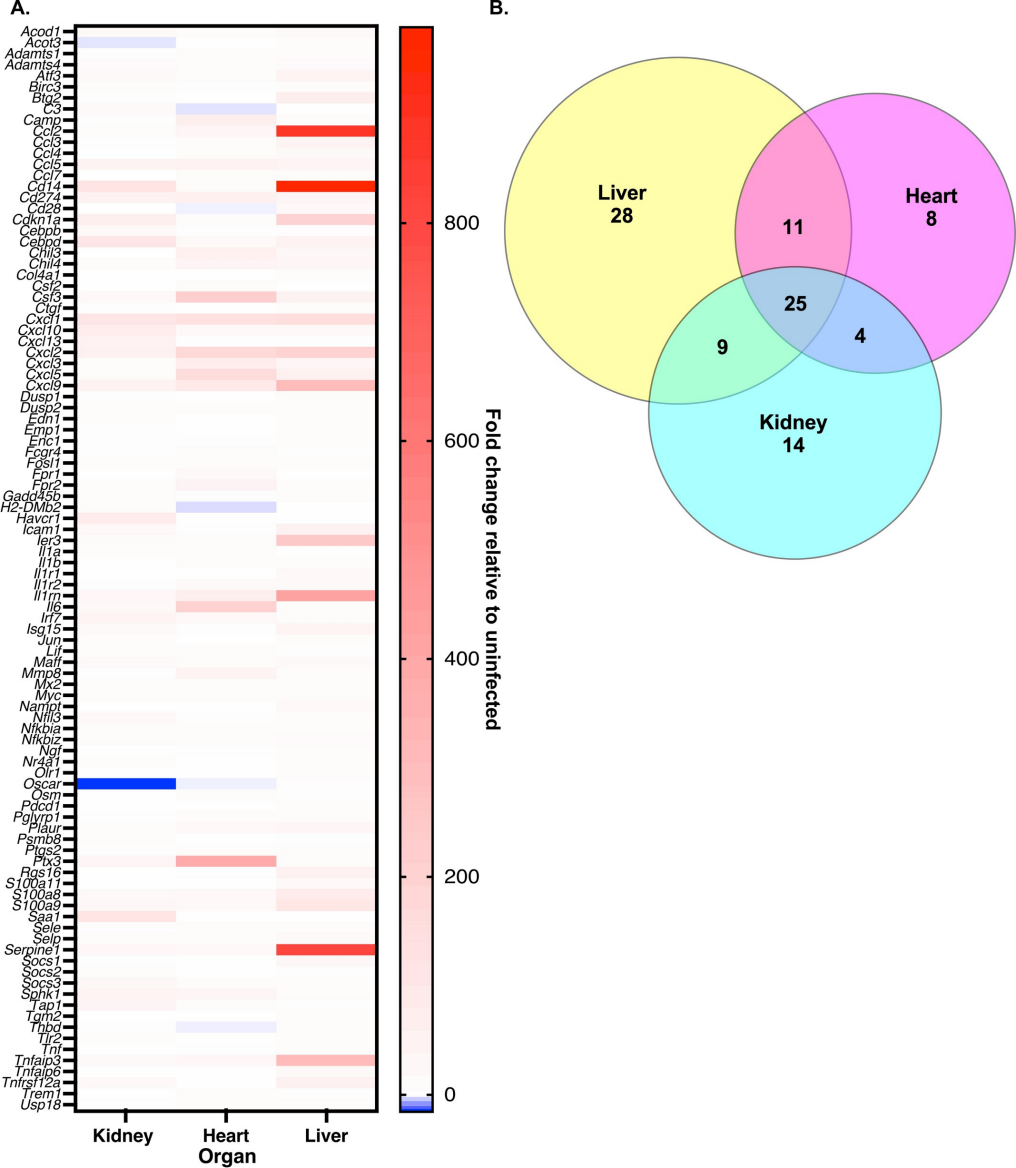
Innate immunity genes commonly associated with Gram-negative bacteremia are upregulated in response to *A*. *baumannii* infection. WT mice were infected systemically with WT *A*. *baumannii* or mock infected with sterile PBS. At 24 h, mice were humanely euthanized, organs were harvested, and RNA was extracted. Gene expression changes in infected versus mock infected mice in the kidney, heart, and liver were determined using NanoString technology and an nCounter mouse Myeloid Innate Immunology Panel (A). Each row represents a different gene from the panel. For clarity only genes that were downregulated ≤ -12-fold or upregulated ≥ +12-fold in at least one organ are shown (top 99 genes with differential expression). A Venn diagram showing the same set of differentially expressed genes as in (A) but highlighting the number of genes in each organ that have altered expression in infected versus mock infected mice, and those that overlap between organs (B).

Upon assessing gene expression changes in WT infected versus mock-infected mice, there was a trend towards broad upregulation of genes associated with the innate immune response with comparatively few genes within the panel downregulated (Fig 1A; S2 Table). Whilst each organ exhibited tissue-specific gene expression changes, 25 shared targets were highly differentially expressed amongst all three organs assessed ((≤ -12-fold or ≥ +12-fold) Fig 1B; S2 Table). Gene ontology (GO) analysis using the Database for Annotation, Visualization, and Integrated Discovery (DAVID) as well as annotation of the NanoString panel revealed that functionally enriched biological processes or GO terms derived from this pool of upregulated genes includes response to cytokines and cytokine stimulus, inflammatory response to infection or injury, defense response against a foreign body or injury, positive regulation of response to external stimulus, response to lipopolysaccharide (LPS), and response to a molecule of bacterial origin (S3 Table) [67–69]. Cell types found to be predominantly associated with these highly expressed genes were neutrophils, which is consistent with the known importance of these cells to controlling *A*. *baumannii* infection [42–46], followed by mast cells and macrophages (S4 Table). All of the aforementioned GO terms and cell-type associations are indicative of activation of the innate immune response to a Gram-negative bacterial pathogen.

Of the genes with highly altered expression in the infected host, both *S100a8* and *S100a9* were detected as being robustly expressed by the Myeloid Innate Immunology panel (Fig 1; S2 Table). Upregulation of these calprotectin subunit-encoding genes during *A*. *baumannii* bacteremia indicates that metal sequestration by the host is likely a crucial factor in controlling growth of the pathogen. In support of this notion, *Il6*, the gene encoding for an essential regulator of metal homeostasis within the host, was also upregulated in all three organs (Fig 1; S2 Table). This proinflammatory cytokine not only induces expression of hepcidin, it also increases expression of the divalent metal transporter ZIP14, which facilitates cellular uptake of metals including manganese, zinc, and non-transferrin-bound iron (S1 and S2 Tables) [70–72]. Notably, the gene encoding for ZIP14 (*Slc39a14*) was also upregulated more than 2-fold in all organs of infected mice. Together these observations suggest a key role for nutritional immunity in *A*. *baumannii* infection.

Other highly upregulated genes during *A*. *baumannii* infection in WT mice included several encoding for proinflammatory cytokines of the chemokine C-X-C (CXC) and C-C (CC) motif families, including CXCL1, CXCL2, CXCL3, CXCL5, CXCL9, CXCL10, CCL2, and CCL5 (Fig 1; S2 Table). Induction of *Cxcl1*, *Cxcl*2, and *Ccl5* is supported by a previous study that determined that expression of these three chemokines is rapidly induced following *A*. *baumannii* systemic infection [44]. Additionally, human neutrophils infected with *A*. *baumannii in vitro* exhibit transcriptional upregulation of *Cxcl10* [73]. The expression of CXCL3, CXCL5, and CXCL9 during *A*. *baumannii* infection has not previously been quantified, and the role that these effectors play in the outcome of infection is unknown. Regardless, the transcriptional induction of predominantly neutrophil chemoattractant chemokines again supports previous studies in mice demonstrating that this cell type plays a critical role in the innate immune response to *A*. *baumannii* infection [42–46].

In contrast to transcriptional overexpression of several chemokines, the receptors for these cytokines, CXCR2 (receptor for CXCL1 through CXCL7) and CXCL3 (receptor for CXCL9, CXCL10, and CXCL11) were not upregulated during *A*. *baumannii* infection. A lack of CXCR2 expression during infection has been linked to neutrophil dysfunction and has been used to delineate between patients with sepsis or infection [74]. Although this correlation is believed to be due to increased internalization of CXCR2, our data suggest that a lack of increased expression of the receptor during bacterial sepsis may also be transcriptionally regulated, however this notion requires further investigation.

Given that the Myeloid Innate Immunology panel is comprised predominantly of targets that would be expected to be expressed during inflammation [67], it is perhaps unsurprising that few transcripts in this array were robustly downregulated during *A*. *baumannii* infection (Fig 1). In fact, the only targets that were downregulated more than 2-fold in all three organs were *Cx3cr1* (-2.0 to -6.6-fold), *Dpp4* (-2.6 to -9.3-fold) and *Hspg2* (-2.5 to -2.8-fold). Downregulation of *Cx3cr1* expression has previously been linked to sepsis in patients, and exposure to *Escherichia coli*, *S*. *aureus*, or purified LPS can lead to decreased expression by monocytes *ex vivo* [75]. The role of *Dpp4* and *Hspg2* in bacterial infections has not been investigated. The largest overall decrease in the expression of a single gene was seen in the kidney, where the transcript *Oscar* was downregulated 15.8-fold in the infected mice versus mock-infected mice (S3A Fig). *Oscar* encodes for the osteoclast-associated immunoglobulin-like receptor (OSCAR), which is a member of the leukocyte receptor complex [76]. In human cells, OSCAR (hOSCAR) is expressed by all myeloid-derived cells and is reported to participate in initiation of the proinflammatory cascade [77]. Indeed, stimulation of neutrophils by hOSCAR *in vitro* leads these cells to release reactive oxygen species (ROS), myeloperoxidase, and lactoferrin, along with other inflammatory mediators [78]. Although the role of hOSCAR in infection has not been interrogated, it is possible that perturbed neutrophil activation via transcriptional downregulation of *Oscar* could allow *A*. *baumannii* to evade the antibacterial effects of these cells. The host transcriptional profile described above for *A*. *baumannii* bacteremia in an immunocompetent mouse provides valuable insights into the innate immune response to this pathogen and highlights avenues for future investigation.

### Host genes associated with nutritional immunity are upregulated during *A*. *baumannii* infection

To further refine our understanding of the host response to *A*. *baumannii* infection and given that we observed upregulation of genes associated with nutritional immunity using the Myeloid Innate Immunology panel, we decided to delve further into this specific aspect of the innate immune response. To this end, a custom NanoString panel was designed and employed to assess for changes in the transcription of genes predominantly associated with nutritional immunity (for a detailed description of each gene target in the panel, see S1 Table). A large body of literature has demonstrated that *A*. *baumannii* is transcriptionally responsive to nutrient metal sequestration by host metalloproteins such as calprotectin [23–26,57–59], as well as to iron and copper stress, both *in vitro* and *in vivo* [79–92]. We therefore hypothesized that nutritional immunity may play an essential role in the innate immune response to this pathogen and sought to further investigate these processes during infection. WT mice were infected systemically with WT *A*. *baumannii* or mock-infected with PBS and samples were prepared for NanoString, as described above.

**Fig 2:**
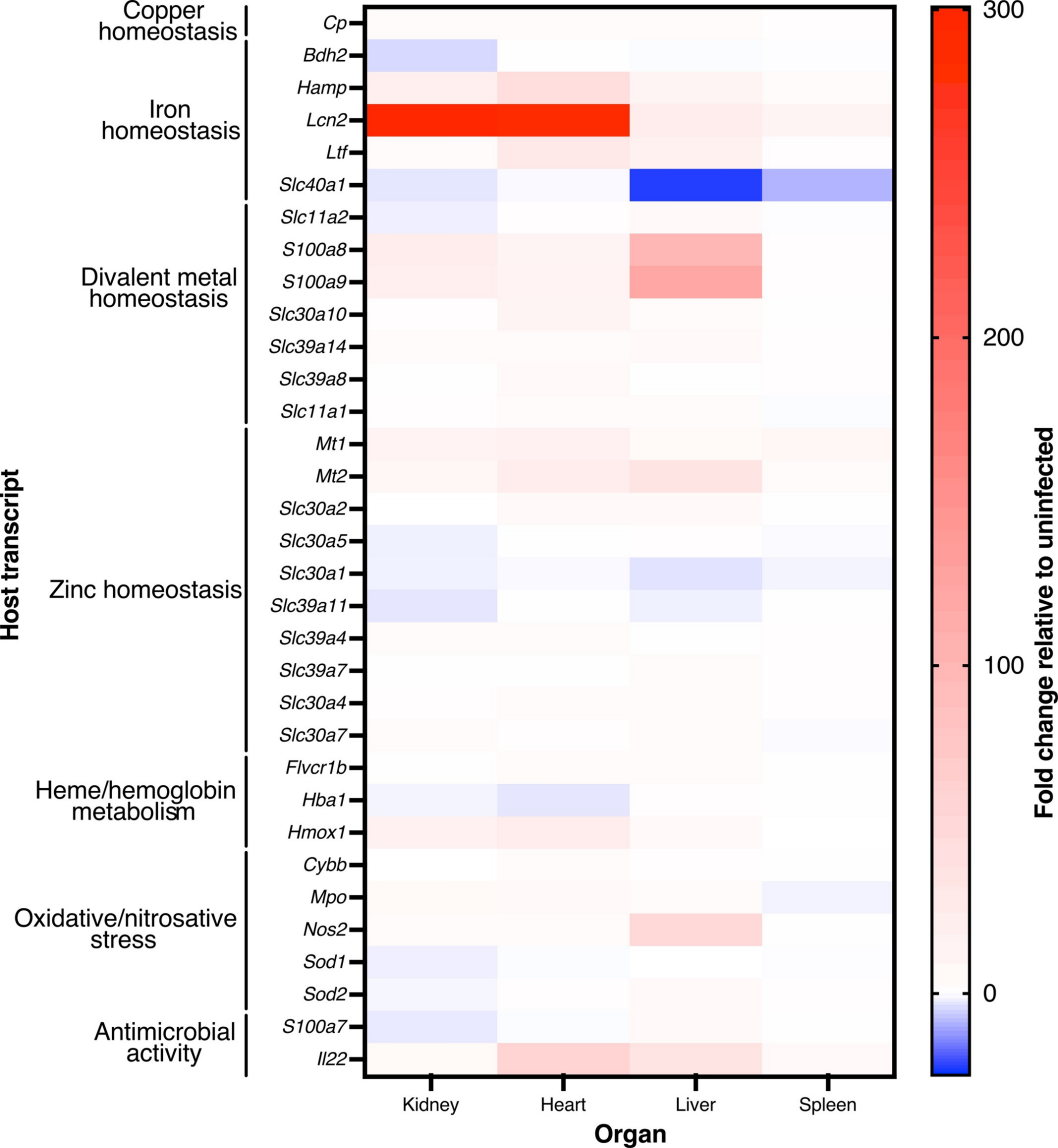
Genes involved in nutritional immunity have altered expression during *A*. *baumannii* infection. Mice were infected systemically with WT *A*. *baumannii* or mock-infected with sterile PBS. At 24 h, mice were humanely euthanized, organs were harvested, and RNA was extracted. The gene expression changes in infected versus mock-infected mice in the kidney, heart, liver, and spleen were determined using NanoString technology and a custom designed code set, as described in the Materials and Methods. Expression was normalized to housekeeping genes *Gapdh*, *Hprt1*, *Pgk1*, and *Tubb*5. Genes are clustered by their primary known or predicted function, as indicated. A description of the function of each gene can be found in S1 Table.

The gene expression changes observed through our targeted NanoString analyses are consistent with the notion that the host undergoes transcriptional reprogramming to induce nutritional immunity during *A*. *baumannii* infection. In validation of the NanoString analyses, both *S100a8* and *S100a9* were detected as being robustly expressed by the targeted array, with comparable fold-changes in the kidneys, heart, and liver to those quantified using the broader Myeloid Innate Immunology panel (Figs 1 and 2), supporting previously reported findings [56]. The genes encoding for both metallothioneins 1 and 2 (*Mt1* and *Mt2*), small cysteine-rich proteins involved in zinc and cadmium sequestration as well as heavy metal detoxification [93,94], were upregulated in all of the organs assessed. Conversely, *Slc30a1* trends towards downregulation in all organs, where this gene encodes for the zinc transporter ZNT1, which is thought to function in the export of zinc from the cytosol to the extracellular milieu [95]. Together, these results suggest that the innate immune response to *A*. *baumannii* infection may involve an effort to restrict extracellular access to free divalent metals such as zinc and manganese.

Whilst genes involved in sequestering or limiting the availability of various transition metals were altered, those exhibiting the greatest transcriptional changes in infected versus mock-infected mice were primarily associated with host iron homeostasis. The process of restricting iron availability during infection is a specific facet of nutritional immunity known as the “hypoferremia of infection/inflammation,” which is predominantly controlled by the peptide hormone, hepcidin [96,97]. Hepcidin is responsible for the internalization and destruction of ferroportin, the only known vertebrate exporter of iron that transports this metal (and others) out of macrophages [98–102]. Hepcidin is encoded for by *Hamp*, whilst ferroportin is encoded for by *Slc40a1*, which were amongst the most highly upregulated and downregulated genes assessed in *A*. *baumannii* infected animals, respectively (Fig 2). Similarly, *Ltf*, which encodes for the iron-sequestering glycoprotein lactoferrin, was robustly expressed in mice infected with *A*. *baumannii* bacteremia (Fig 2). These results indicate that the host likely limits serum availability of iron during *A*. *baumannii* infection by sequestering it within cells or protein complexes.

The gene exhibiting the highest upregulation in our dataset during *A*. *baumannii* infection was *Lcn2* (~12 to 300-fold, Fig 2). As described above, LCN2 is an acute phase protein that functions as a bacteriostatic agent by binding primarily catecholate and certain mixed-type siderophores, thereby directly impeding iron acquisition by invading bacteria [62–64]. Additionally, LCN2 binds to the mammalian siderophore 2,5-dihydroxybenzoic acid (2,5-DHBA) which may further help to reduce bioavailable iron and maintain host iron homeostasis [103]. These results indicate that nutritional immunity, and specifically LCN2, may be playing key roles in the host innate immune response against *A*. *baumannii* infection.

### LCN2 is robustly expressed in *A*. *baumannii* infected tissues

**Fig 3:**
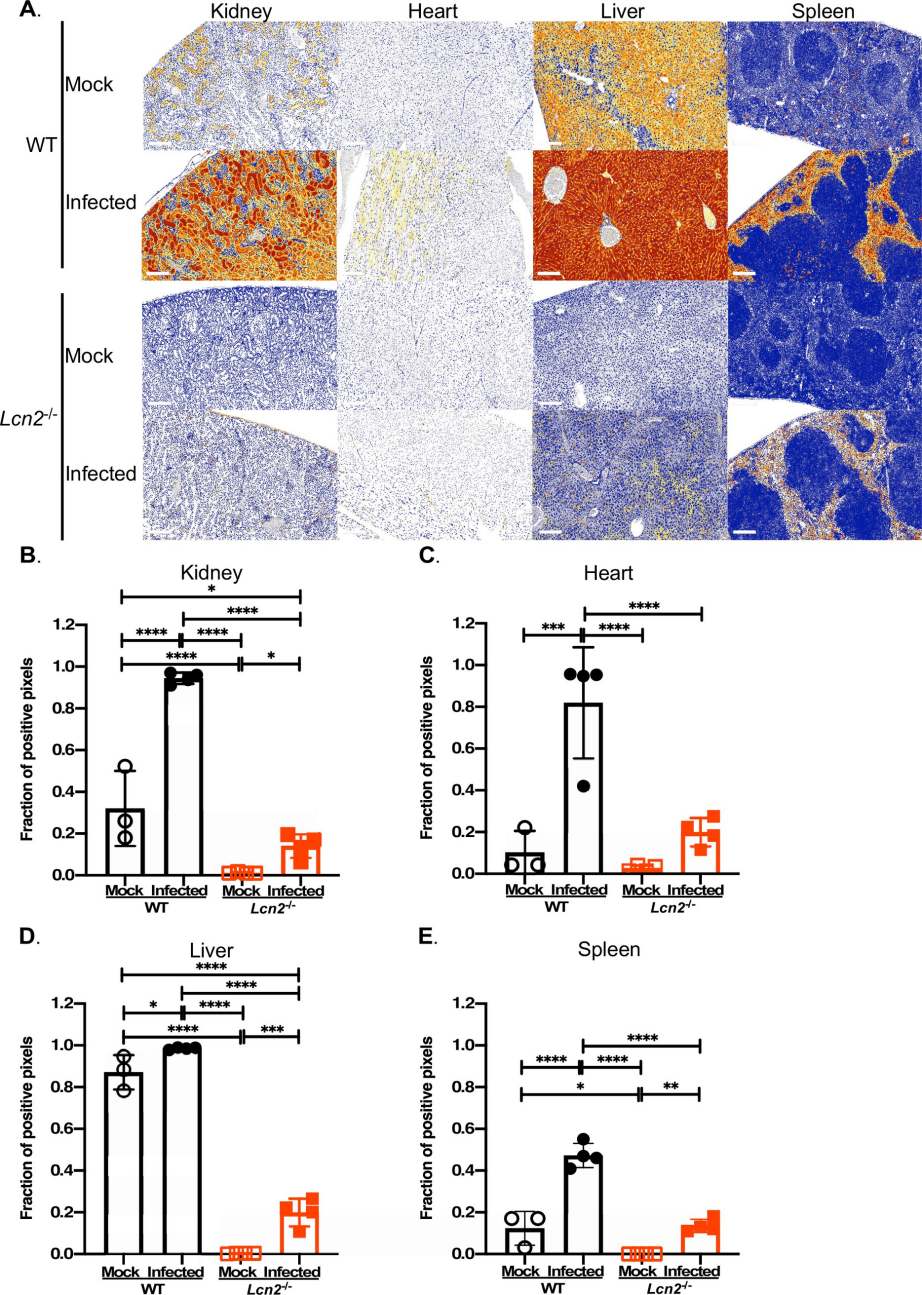
LCN2 is expressed in the *A*. *baumannii* infected host. WT or *Lcn2*^*-/-*^ knockout mice were infected systemically with WT *A*. *baumannii* or mock-infected with sterile PBS. After 24 h, the mice were humanely euthanized, the organs were harvested and subsequently fixed using 10% neutral buffered formalin. Tissues were sectioned and LCN2 was detected using polyclonal goat ɑ-mouse LCN2 antibody and ɑ-goat IgG. Immunolabeling was detected with 3,3’-diaminobenzidine (DAB) and slides were counterstained with stained with hematoxylin. Whole slide images were false-colored to represent four expression levels for LCN2, as indicated by the legend (0+ blue, 1+ yellow, 2+ orange, 3+ red) (A). Representative photomicrographs from three mice per group are shown. The scale bar represents 200 μM. Digital tissue image analyses were performed to determine the fraction of LCN2 positive pixels relative to total pixels in the whole tissue section (B-E). Each symbol represents data collected from an individual mouse. Statistical analysis was performed by one-way ANOVA using Holm-Sidak’s multiple comparisons post-test where *p < 0.05, ** p < 0.01, *** p < 0.001 and **** p < 0.0001.

Given the strong upregulation of *Lcn2* in the *A*. *baumannii* infected host during bacteremia, we sought to determine if this translates to increased production of the LCN2 protein *in vivo*. WT or *Lcn2-*deficient (*Lcn2*^*-/-*^) mice were systemically infected with *A*. *baumannii* or mock-infected, as described above [63]. The kidneys, hearts, livers, and spleens were harvested after 24 h, LCN2 was detected by immunohistochemistry (IHC) using fixed and sectioned tissue samples, and the relative magnitude of protein expression was quantified using automated digital tissue image analysis. In WT uninfected animals, LCN2 immunolabeling was observed in each organ highlighting that basal LCN2 expression exists in the absence of *A*. *baumannii* infection (Fig 3; S5 through S8 Tables). Consistent with the transcriptomics results, infection of WT mice with *A*. *baumannii* resulted in broad, multiorgan and multicellular increases in LCN2 immunolabeling, with distinct patterns of localization. In the kidneys of mock-infected mice, moderate overall LCN2 staining was observed, which localized to the apical membrane of the cortical convoluted tubules and increased upon *A*. *baumannii* infection (Fig 3A and 3B; S5 Table). Negligible staining of cardiac LCN2 was observed in WT uninfected mice (Fig 3A and 3C; S6 Table), whereas infected mice exhibited low to moderate LCN2 staining localized to cardiomyocytes, and very high staining localized within interstitial macrophages. Basal immunolabeling of LCN2 in uninfected livers was high, and a trend existed towards increased detection of the protein as distance from the central vein within a hepatic lobule increased (Fig 3A and 3D; S7 Table) [104]. During infection, very high staining of LCN2 was detected in the livers, which localized with hepatocytes throughout the lobule. Scattered high level LCN2 staining was also colocalized to tissue-resident macrophages (Kupffer cells). Low level LCN2 immunolabeling was detected overall in the spleens, where infection with *A*. *baumannii* lead to moderate expression, which localized with red pulp myeloid cells (Fig 3A and 3E; S8 Table). No notable immunolabeling of LCN2 was observed in any organ of the mock-infected *Lcn2*^*-/-*^ mice (Fig 3), confirming the known abolishment of *Lcn2* gene expression and global LCN2-deficiency in these animals [63]. There was, however, low levels of LCN2 staining detected in the organs of infected *Lcn2*-deficient mice, suggesting the existence of a bacterial antigen or induction of an infection-responsive host protein that is cross-reactive with the α-LCN2 antibody. The specificity of the antibody was tested through immunoblotting, using organ homogenates from infected and mock-infected mice, and whole cell lysates (WCLs) from the bacteria. *A*. *baumannii* was grown under iron-replete and deplete conditions, the latter being representative of the iron-restricted host environment. Although a non-specific band at ~12–15 kDa was observed in some samples derived from WT infected animals (predominantly in the kidneys), no such cross-reactivity was evident in any of the organ homogenates from infected *Lcn2*^*-/-*^ mice (S1 Fig). Additionally, no immunoreactivity was observed in the immunoblots performed using the bacterial WCLs and the α-LCN2 antibody, regardless of iron status (S2 and S3 Figs). The identity of the cross-reactive antigen(s) remains unknown, and is the subject of further investigation. Altogether these findings parallel the observation of increased expression of *Lcn2* during infection, elevated serum LCN2 during sepsis [105], and further support a possible role for LCN2 in the innate immune response to *A*. *baumannii*.

### Differences in infection outcomes between WT and *Lcn2-*deficient mice cannot be readily explained by transcriptional changes

To begin characterizing how the loss of LCN2 might impact the inflammatory response to *A*. *baumannii* infection, we also investigated gene expression changes in WT versus *Lcn2*-deficient mice via NanoString. Relative to the gene expression changes observed between WT infected versus mock-infected animals (Fig 1), very few of the targets assessed were differentially expressed between infected WT and *Lcn2*-deficient mice (S4 Fig). These results suggest that loss of *Lcn2* expression does not lead to broad overall transcriptional changes within the host. Although the loss of *Lcn2* was systemic, no transcript was differentially expressed more than ± 2-fold in all three organs, suggesting that what transcriptional changes do occur are largely organ specific (S4 Fig). For example, while the expression of *S100a8* and *S100a9* was unchanged or trended towards downregulation in the heart and liver, both genes were transcriptionally upregulated in the *Lcn2*^*-/-*^ kidney, suggesting a potential compensatory mechanism for perturbed metal homeostasis in the *Lcn2*-deficient mice in this organ. In the heart, *cxcl13* was the most highly upregulated gene in the *Lcn2*-deficient mice, and this target was elevated in the liver as well. CXCL13 is a B cell attracting chemokine and while its role in *A*. *baumannii* infection is unclear, it has previously been identified as a biomarker for sepsis and a predictor of mortality in COVID-19 patients [106–108]. Whether or not increased transcription of *cxcl13* results in elevated expression of the chemokine during infection is unknown, as is its potential role in *A*. *baumannii* sepsis. Regardless, alterations in the transcription of innate immunity genes in the *Lcn2*-deficient mice versus WT mice do not readily reveal how LCN2 contributes to *A*. *baumannii* infection; a role that may be multifactorial and impact metal homeostasis, immune cell populations and/or recruitment, or the production of inflammatory mediators [109].

### LCN2 inhibits iron acquisition by *A*. *baumannii in vitro*

Given the apparent induction of the hypoferremia of infection/inflammation by *A*. *baumannii* within the host (Figs 1 and 2; S2 Table), further highlighting the importance of iron during infection, we next sought to elucidate if LCN2 can inhibit acquisition of this metal by *A*. *baumannii*. The strain *A*. *baumannii* ATCC 17978 encodes for three different families of ten discrete siderophores: fimsbactins A through F, which are mixed type siderophores possessing both mono-hydroxamate and bis-catecholate moieties [110], the mixed type mono-hydroxamate and mono-catecholate siderophores acinetobactin and pre-acinetobactin [111–113], and the hydroxamate siderophores baumannoferrins A and B [114]. Although LCN2 is unlikely to coordinate hydroxamate siderophores such as the baumannoferrins, due to structural incompatibility of these molecules with the LCN2 calyx [62], it is capable of sequestering specific mixed type siderophores [63,64,115,116]. To date, however, the interaction between LCN2 and *A*. *baumannii* siderophores has not been investigated.

**Fig 4:**
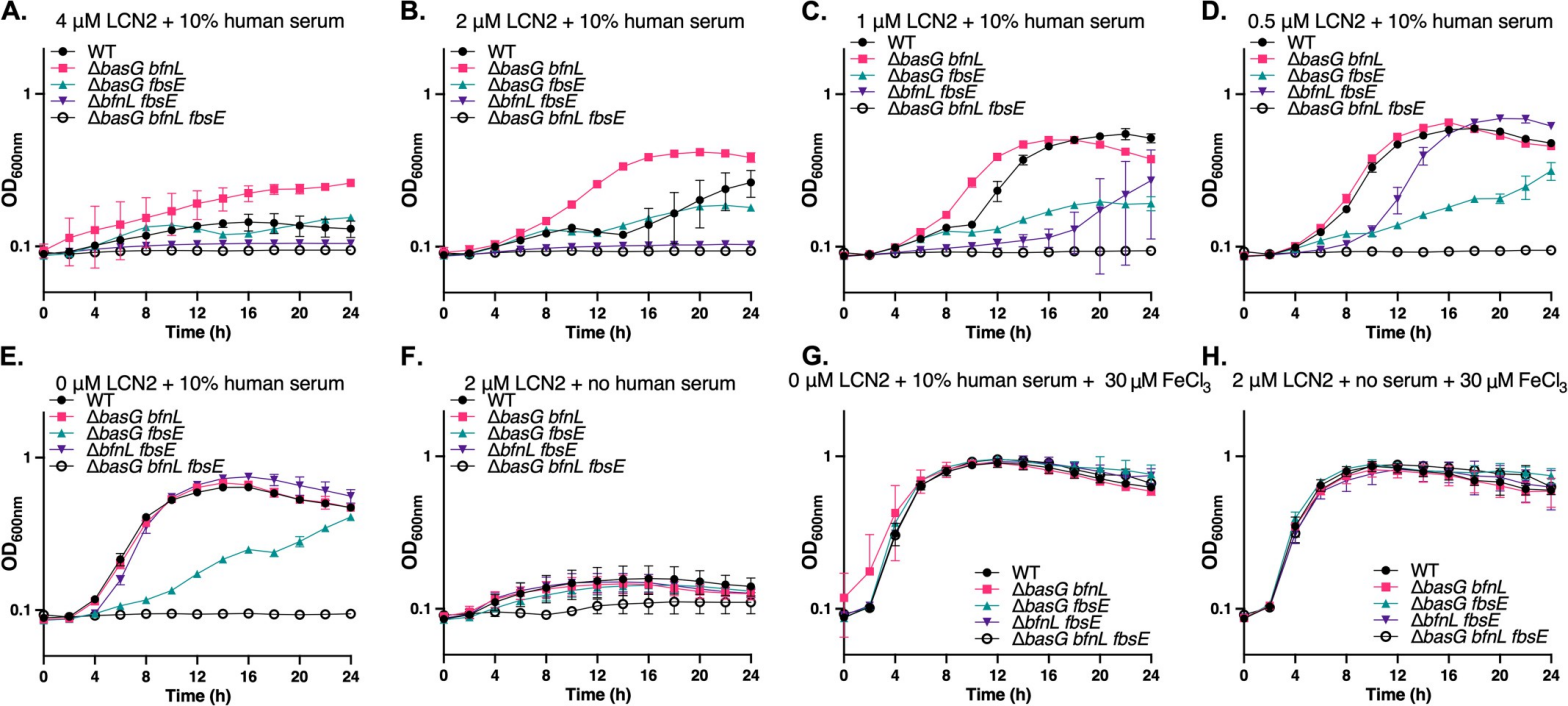
Recombinant LCN2 inhibits the iron-dependent growth of *A*. *baumannii in vitro*. WT *A*. *baumannii* and its isogenic siderophore biosynthetic mutants were grown in iron-restricted cTMS media and included strains proficient in the production of fimsbactins only (Δ*basG bfnL*), baumannoferrin only (Δ*basG fbsE*), acinetobactin only (Δ*bfnL fbsE*), or disrupted for biosynthesis of all three siderophore families (Δ*basG bfnL fbsE*). WT is proficient in the production of all three siderophore families. The strains were grown in cTMS with 10% human serum and various concentrations of recombinant LCN2 (A-E), 2 μM LCN2 without serum (F), 0 μM LCN2 with 10% serum and exogenous FeCl_3_ (G), or 2 μM LCN2 without serum but with exogenous FeCl_3_ (H). Bacterial growth was assessed by determining the OD_600nm_ over 24 h. Data are representative of two experiments performed in biological triplicate and error bars represent the standard error of the mean.

To interrogate the possible sequestration of *A*. *baumannii* siderophores by LCN2 and to assess the impact on bacterial growth, a panel of previously generated siderophore biosynthetic mutants was employed [19]. These strains include WT *A*. *baumannii* which encodes for all three families of siderophores, Δ*basG bfnL* which encodes for the fimsbactins alone, Δ*basG fbsE* which encodes for only baumannoferrins A and B, Δ*bfnL fbsE* which encodes for acinetobactin and pre-acinetobactin, and a complete siderophore biosynthetic knockout Δ*basG bfnL fbsE* (Table 1). By utilizing strains expressing only one siderophore, it should be possible to ascertain which, if any, are able to evade capture by LCN2 and thus facilitate iron acquisition by *A*. *baumannii*. The aforementioned strains of bacteria were grown in chelex-treated Tris minimal succinate media (cTMS [117]) supplemented with 10% human serum as the sole iron source. Although any residual free iron in the metal-deplete cTMS media should be sequestered by serum transferrin, siderophores are capable of pillaging iron from the host glycoprotein and thus are required for growth under these conditions. LCN2 was added to the media at varying concentrations as previously described [63], and bacterial growth was assessed by determining optical density at 600 nm (OD_600nm_) over 24 h. Interestingly, the growth of all strains of *A*. *baumannii* was impaired in the presence of 4 μM of LCN2 (Fig 4A), including the siderophore-proficient WT, indicating that the presence of high concentrations of LCN2 can inhibit the iron-dependent growth of *A*. *baumannii*. As expected and previously observed, the strain disrupted for biosynthesis of all three siderophore families was unable to grow under these conditions [19]. At 4 μM and 2 μM of LCN2 (Fig 4A and 4B), the strain expressing acinetobactin alone (Δ*bfnL fbsE*) was also unable to grow and was inhibited for growth at lower concentrations of LCN2 (Fig 4C and 4D). These results suggest that iron acquisition facilitated by acinetobactin is hindered in the presence of LCN2, either through sequestration by the protein or through an additional undetermined mechanism. By contrast, the strain expressing fimsbactins alone grew as well as, or better than WT, where both strains exhibited a dose-dependent growth enhancement with decreasing LCN2 concentrations (Fig 4A through 4E). We previously demonstrated that a strain expressing only fimsbactins is capable of growing on serum as a sole iron source in a manner comparable to WT [19]. However, the ability of Δ*basG bfnL* to grow in the presence of both serum and LCN2 suggests that one or more of the fimsbactins are not bound by the LCN2 and/or that in the absence of the other siderophores are produced at sufficiently high amounts to overwhelm sequestration. Supporting the notion that fimsbactins are required for growth in the presence of LCN2, a small but reproducible growth defect was observed when a mutant defective in fimsbactins production (Δ*fbsE*) was grown in the presence of LCN2 alone (S5 Fig). Similar growth defects were not noted for biosynthetic mutants of acinetobactin (Δ*basG*) or baumannoferrins (Δ*bfnL*) relative to WT. Lastly, the strain producing the baumannoferrins alone (Δ*basG fbsE*) was attenuated for growth in cTMS media with serum regardless of the presence of LCN2 (Fig 4A through 4E). These findings recapitulate our previous observation that Δ*basG fbsE* grows poorly in the presence of serum [19], and the known lack of binding affinity for hydroxamate siderophores by LCN2 [63,64,115,116]. All other strains were capable of growing comparably to WT on 10% serum as a sole iron source in the absence of LCN2, except for the siderophore-deficient mutant (Fig 4E [19]), whereas in the absence of human serum all strains grew poorly (Fig 4F). The effects observed were confirmed to be iron-dependent as the addition of excess exogenous iron restored growth of all strains (Fig 4G and 4H). Together these results indicate that iron is mobilized from serum transferrin in the presence of LCN2 to support *A*. *baumannii* growth, and that siderophores are essential to this process. Further, our data suggest that the fimsbactins may be better able to evade sequestration by LCN2 *in vitro* than acinetobactin and/or pre-acinetobactin. Collectively, these findings support the hypothesis that the robust expression of LCN2 during infection may be a countermeasure employed by the host to restrict bacterial survival, proliferation, or dissemination by inhibiting iron acquisition by *A*. *baumannii*.

**Table 1:** Strains employed in this study.

Strain	Description^1^	Reference
*A*. *baumannii* ATCC 17978 UN	WT *A*. *baumannii* from fatal pediatric meningitis, UN isolate. Reference genome CP079931.	[172,174]
*A*. *baumannii* ATCC 17978 VU	WT *A*. *baumannii* from fatal pediatric meningitis, VU isolate. Reference genomes NZ_CP018664; CP012004	[172,174–176]
Δ*basG*	Acinetobactin and pre-acinetobactin biosynthetic mutant; markerless	[19]
Δ*bfnL*	Baumannoferrin A and B biosynthetic mutant; markerless	[19]
Δ*fbsE*	Fimsbactins A through F biosynthetic mutant; markerless	[19]
Δ*basG bfnL*	Acinetobactin and baumannoferrins biosynthetic mutant. Encodes for the fimsbactins; Kan^R^	[19]
Δ*basG fbsE*	Acinetobactin and fimsbactins biosynthetic mutant. Encodes for the baumannoferrins; Kan^R^	[19]
Δ*bfnL fbsE*	Baumannoferrins and fimsbactins biosynthetic mutant. Encodes for acinetobactin and pre-acinetobactin; Kan^R^	[19]
Δ*basG bfnL fbsE*	Acinetobactin and pre-acinetobactin, baumannoferrins, and fimsbactins biosynthetic mutant. Siderophore-deficient; Kan^R^	[19]
WT *A*. *baumannii* VU/ p.P_*fbsB*_.*luxABCDE*.MU368.*tet*	WT *A*. *baumannii* containing the p.*luxABCDE*.MU368.*tet* plasmid with the iron-regulated *fbsB* promoter cloned upstream of *luxABCDE*; Tet^R^	This study
WT *A*. *baumannii* VU/ p.*luxABCDE*.MU368.*tet*	WT *A*. *baumannii* containing the promoterless p.*luxABCDE*.MU368.*tet* plasmid construct; Tet^R^	[25]

^1^Strains marked Kan^R^ have the last gene in their strain designation replaced with a kanamycin resistance determinant.

### LCN2 induces iron-regulated gene expression in *A*. *baumannii*

**Fig 5:**
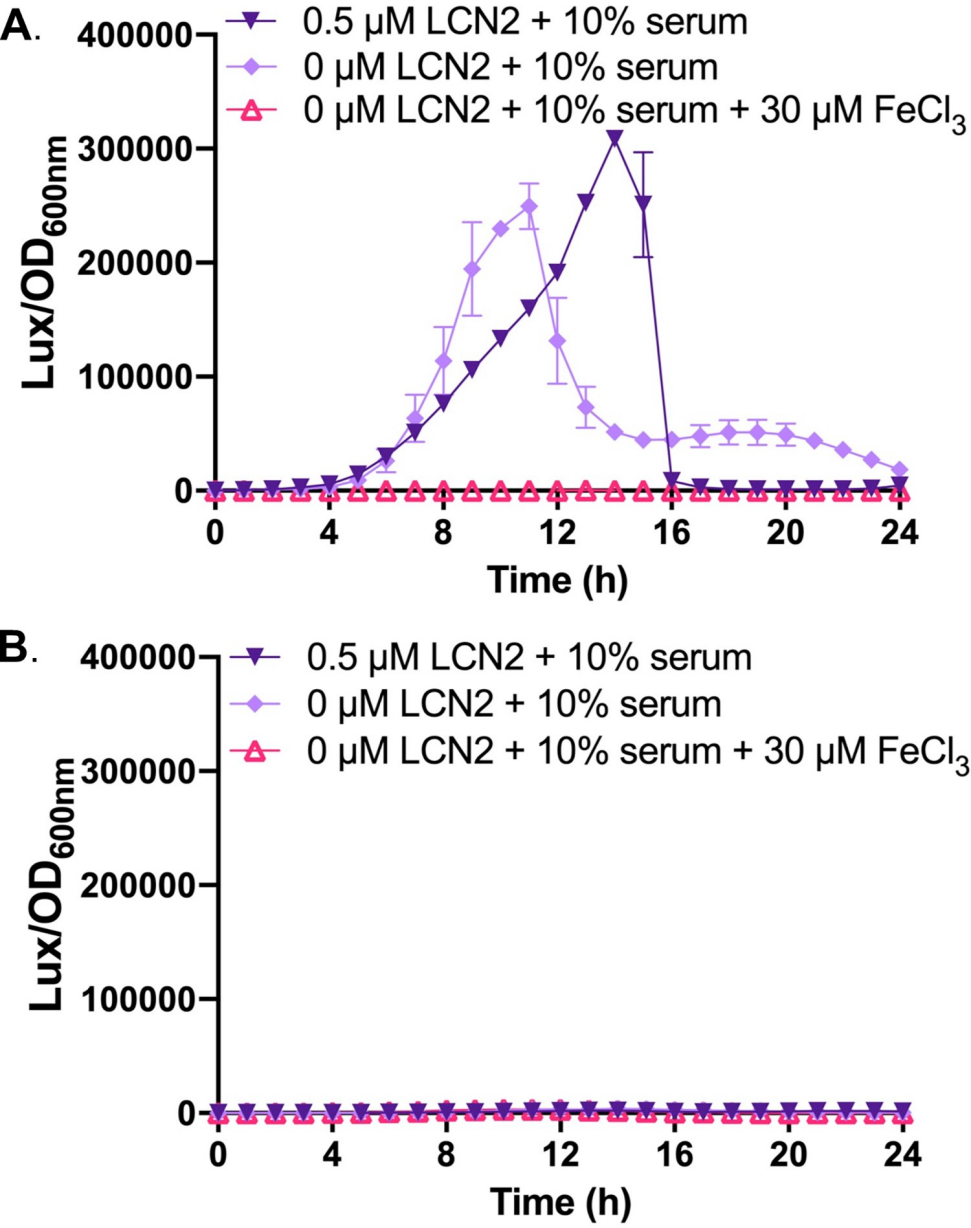
Recombinant LCN2 induces iron-regulated gene expression in *A*. *baumannii*. WT *A*. *baumannii* possessing a p.P_*fbsB*_.*luxABCDE*.MU368.*tet* reporter construct (A) or the promoterless p.*luxABCDE*.MU368.*tet* plasmid (B) were grown in iron-restricted cTMS media with 10% serum and the indicated concentrations of recombinant LCN2 or exogenous FeCl_3_. Bacterial growth was assessed by determining the OD_600nm_ and gene expression was assessed through luciferase activity over 24 h. Relative luminescence units (LUX) are expressed as LUX/OD_600nm_ over time. Data are representative of two experiments performed in biological triplicate and error bars represent standard error of the mean.

To further confirm that LCN2 inhibits *A*. *baumannii* growth through iron sequestration, we sought to determine if the presence of LCN2 induces iron-regulated gene expression in the bacteria *in vitro*. A reporter construct was generated such that the promoter of a known iron-responsive gene was used to drive the expression of luciferase (Table 1). In this case, the promoter for the fimsbactins biosynthesis gene *fbsB* was selected, as it was previously found to be the most highly upregulated gene in *A*. *baumannii* during iron starvation [19]. WT *A*. *baumannii* containing either the reporter construct (p.P_*fbsB*_*luxABCDE*.MU368.*tet*) or the promoterless vector (p.*luxABCDE*.MU368.*tet*) were grown under iron restriction with and without the addition of recombinant murine LCN2, as described above. LCN2 robustly induces *fbsB*-driven luciferase expression beyond induction with just 10% human serum alone, whereas the addition of exogenous iron abolished luciferase activity (Fig 5A). No luminescence was detected using the promoterless construct under any condition tested (Fig 5B). These results confirm that gene expression from the *fbsB* promoter is inhibited by iron, and further indicate that LCN2 induces iron starvation in *A*. *baumannii* likely through sequestration of one or more siderophores.

### LCN2 is not required to control bacterial survival and proliferation during *A*. *baumannii* bacteremia but is protective against mortality

**Fig 6:**
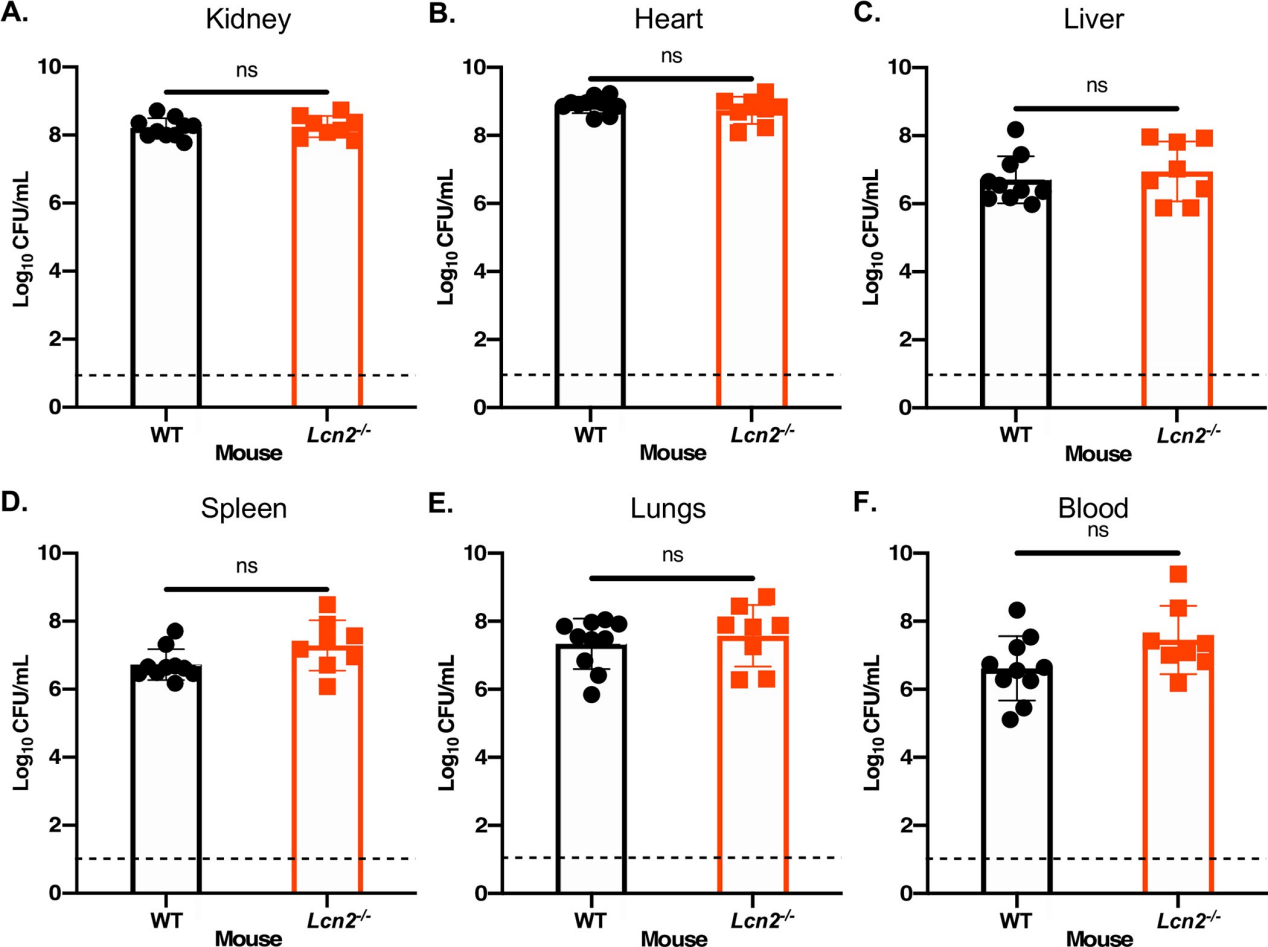
Mice deficient in *Lcn2* expression have comparable bacterial burdens to WT mice following *A*. *baumannii* bacteremia. *Lcn2*^*-/-*^ knockout mice and their WT C57BL/6 littermate controls were infected retro-orbitally with WT *A*. *baumannii*. The infection was allowed to proceed for 24 h before the mice were humanely euthanized and the organs were harvested. Organs were homogenized, serially diluted in PBS and plated to Luria-Bertani agar (LBA). The bacterial burdens of the kidneys (A), heart (B), liver (C), spleen (D), lungs (E) and blood (F) were determined. Each symbol represents the WT *A*. *baumannii* count in the corresponding organ of one animal. Data are compiled from two independent experiments. Statistical significance was determined by Mann-Whitney *U* test, where ns = not significant. The dashed line represents the limit of detection for the assay.

**Fig 7:**
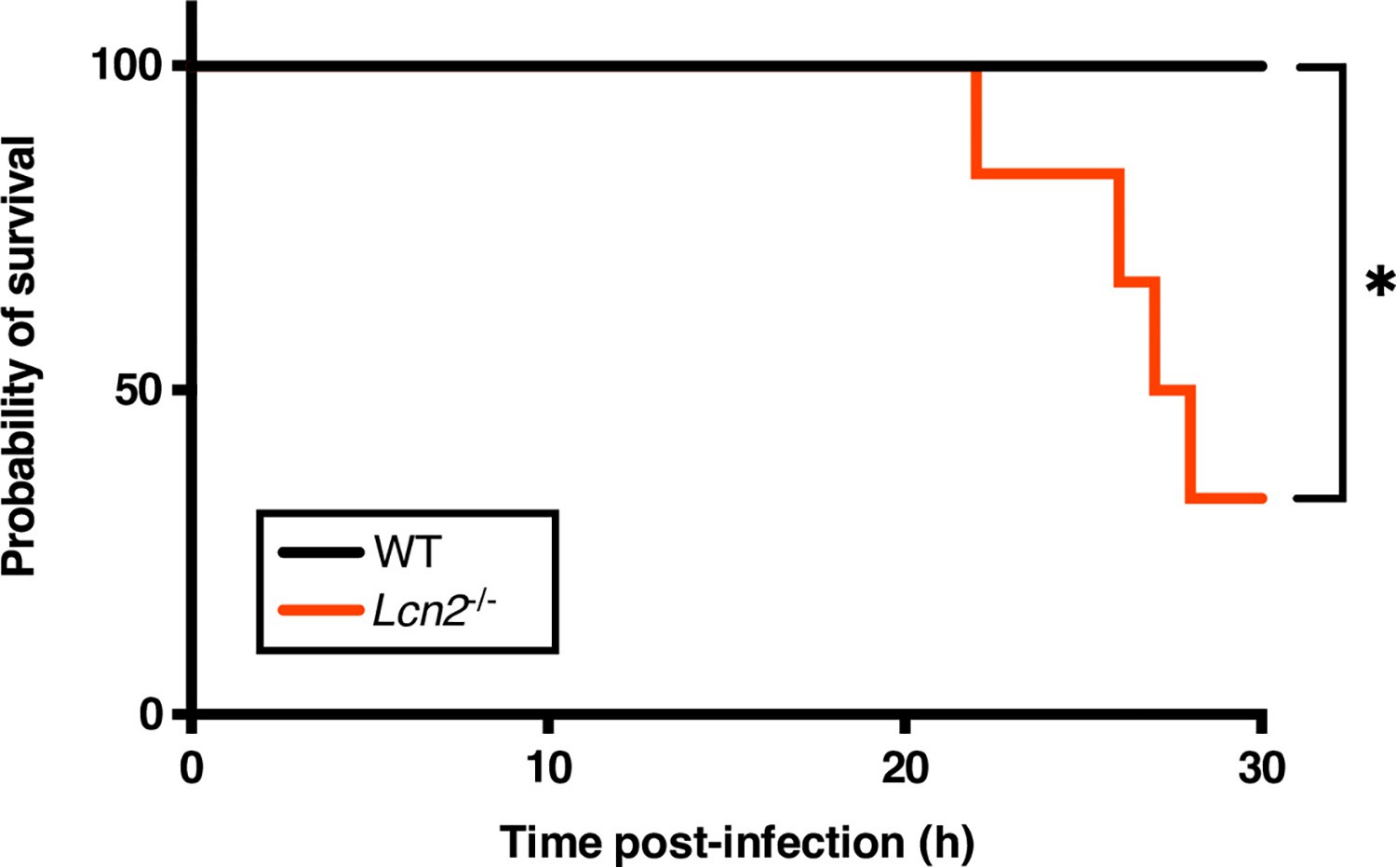
LCN2-deficient mice exhibit increased mortality from *A*. *baumannii* bacteremia. *Lcn2*^*-/-*^ knockout mice and their WT C57BL/6 littermate controls were infected retro-orbitally with a sublethal dose of WT *A*. *baumannii*. Mice were sacrificed upon meeting humane endpoint criteria. Statistical significance was determined by Mantel-Cox test where *p < 0.05; n = 6.

Having confirmed that both the *Lcn2* transcript and corresponding protein increase during *A*. bacteremia (Figs 2 and 3 and S1 Fig; S5 through S8 Tables), and that LCN2 impacts iron homeostasis by the bacteria *in vitro* (Figs 4 and 5), we endeavored to determine the overall influence of this host innate immune protein on infection. To this end, *Lcn2*-deficient mice [63] and their WT littermate controls were infected with a non-lethal dose of WT *A*. *baumannii* (2 x 10^8^–5 x 10^8^ CFU), as described above. After 24 h of infection followed by humane euthanasia, the organs were harvested, homogenized, and bacterial burdens were determined. Despite a slight trend towards increased bacterial burdens in the *Lcn2*^-/-^ mice, these burdens did not differ significantly between mouse genotypes in any of the organs or the blood during infection (Fig 6). We observed that the *Lcn2*-deficient mice did, however, appear to exhibit signs of increased disease severity (e.g. hunched posture, lethargy, ruffled fur [118]), relative to WT mice. To address the possibility that *Lcn2* may impact the outcome of infection independent of controlling bacterial burdens during bacteremia, and to determine the impact of *Lcn2* expression during an acute sublethal infection, we increased the inoculum used to infect the WT and *Lcn2*-deficient mice from 2 x 10^8^–5 x 10^8^ CFU, to 2 x 10^9^ CFU (~1 log increase). Mice were challenged systemically with this infectious dose and monitored for disease severity over the course of infection. Consistent with the observation that the *Lcn2*-deficient mice exhibited more severe symptoms of infection when employing the lower infectious dose, we found that 60 percent of these mice met humane endpoint criteria by 30 h [119], whilst none of the WT mice required euthanasia at this timepoint (Fig 7). No additional mice met endpoint criteria in the monitoring period after 30 h. The impact of LCN2 on the pathophysiology of *A*. *baumannii* bacteremia appears to function independent of restricting bacterial growth through iron and/or siderophore sequestration under the conditions tested and may be multifactorial.

### *Lcn2* is required to control survival and dissemination of WT *A*. *baumannii* during pneumonia

**Fig 8:**
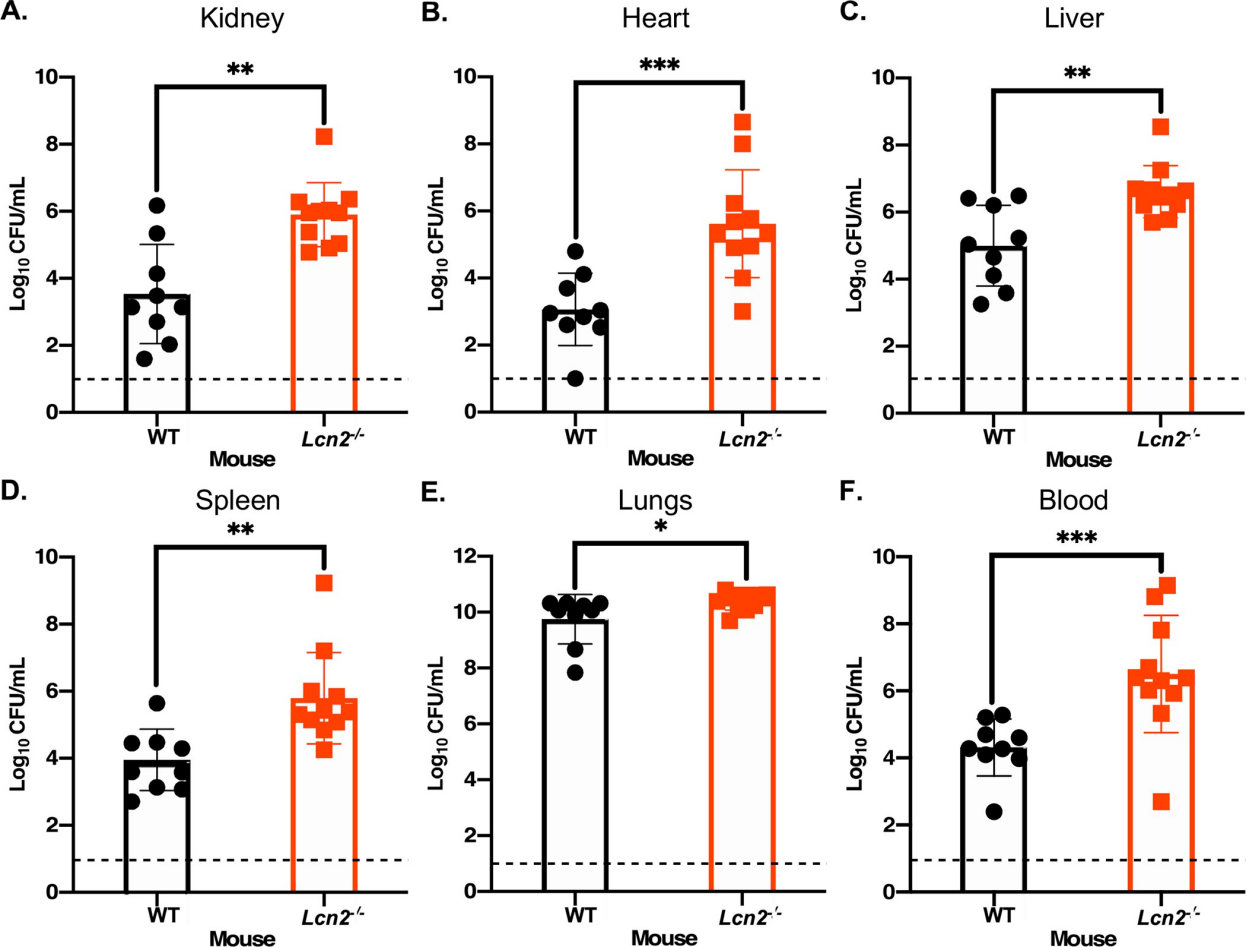
Mice deficient in LCN2 expression have increased susceptibility to *A*. *baumannii* pneumonia. *Lcn2*^*-/-*^ knockout mice and their WT C57BL/6 littermate controls were infected intranasally with WT *A*. *baumannii*. The infection was allowed to proceed for 36 h before the mice were humanely euthanized and the organs were harvested. Organs were homogenized, serially diluted in PBS and plated to LBA. The bacterial burdens of the kidneys (A), heart (B), liver (C), spleen (D), lungs (E), and blood (F) were determined. Each symbol represents the WT *A*. *baumannii* count in the corresponding organ of one animal. Data are compiled from two independent experiments. Statistical significance was determined by Mann-Whitney *U* test, where *p < 0.05, ** p < 0.01, and *** p < 0.001. The dashed line represents the limit of detection for the assay.

As VAP and bacteremia are the most frequent and formidable presentations of *A*. *baumannii* infection [120], the role of *Lcn2* in a murine model of pneumonia was also assessed. WT and *Lcn2*-deficient mice were infected intranasally with 2 x 10^8^–4 x 10^8^ CFU of WT *A*. *baumannii*. The infection was allowed to proceed for 36 h before the mice were humanely euthanized, the organs harvested, and bacterial burdens determined. Unlike in the murine model of bacteremia, the bacterial burdens recovered from *Lcn2*-deficient mice relative to their WT counterparts were significantly higher in every organ assessed, as well as the blood, by ~1.5 to 2.8-log_10_ (Fig 8). Notably, the bacterial burdens in the lungs in *Lcn2*-deficient mice exceeded the original inoculum, suggesting that these mice failed to constrain bacterial replication and the increased burdens in the organs and blood indicates a failure of the *Lcn2*^*-/-*^ mice to prevent survival and/or extrapulmonary dissemination of *A*. *baumannii* in the host. The results from these *in vivo* studies reveal that LCN2 has differing impacts on the outcome of *A*. *baumannii* bacteremia and pneumonia in mice, suggesting a complex role in the innate immune response to this pathogen.

## Discussion

The threat posed by multidrug-resistant *A*. *baumannii* was already of particular concern to the global health community before 2019 [9,10], however this risk has been further exacerbated by the ongoing COVID-19 pandemic [11,12]. Both the increased number of immunocompromised mechanically-ventilated patients and the overuse of antibiotics have created an environment which may drive further development of antimicrobial resistance in *A*. *baumannii* and other pathogens that are already extensively drug resistant [16]. Pan-resistant strains of *A*. *baumannii* have been reported since the early 2000s [121], and their prevalence has increased steadily ever since first being documented [122]. In the case of extensively and pandrug-resistant *A*. *baumannii*, therapeutic options are limited largely to expensive synergistic combination therapies with lower efficacy, higher levels of toxicity, and more severe side effects [122,123]. Because of this, there is a heightened need for accelerated efforts to identify new and unconventional therapeutic strategies in the treatment of *A*. *baumannii* infections. Treatment approaches that augment the host immune response are particularly attractive given the propensity of *A*. *baumannii* and other ESKAPE pathogens to infect immunocompromised individuals [36–39]. Additionally, exploiting metal availability to the pathogen also represents a promising avenue for drug development, given the essentiality of nutrient metals to bacterial proliferation. The success of this approach is exemplified by the most recently approved antibiotic in the treatment of *A*. *baumannii*, a siderophore-antibiotic conjugate known as Cefiderocol, that uses a “Trojan horse”-like strategy to deliver its toxic payload intracellularly by exploiting native bacterial transporters for iron acquisition [124–126]. Here we further characterize the host innate immune response to *A*. *baumannii* infection to identify potential avenues for future therapeutic development, with a particular focus on facets of nutritional immunity. We demonstrate that nutritional immunity is robustly induced during *A*. *baumannii* bacteremia, and that LCN2 is an essential part of this response. LCN2 is highly expressed *in vivo*, and in its absence infection outcomes are exacerbated in murine models of both *A*. *baumannii* bacteremia and pneumonia. *In vitro*, LCN2 restricts the iron-dependent growth of *A*. *baumannii* and induces iron-regulated gene expression, suggesting that at least one of the siderophores required for iron acquisition by the bacterium is sequestered by the acute phase protein. These findings highlight that nutritional immunity is an important aspect of the host innate immune response in controlling *A*. *baumannii* infection and identify LCN2 as a promising target for drug development in the treatment of these infections.

The effects of LCN2 on the outcome of infection has been interrogated for a handful of pathogens using *Lcn2*-deficient mice [63,105,127–135]. *Lcn2* and its encoded protein are strongly upregulated in mammals in response to exposure to purified LPS, Gram-negative bacteria, mycobacteria, and various parasites, and the effects of LCN2 deficiency appear to be pleiotropic and dependent upon the pathogen and route of infection [63,105,127–135]. *Lcn2*-deficient mice have increased intracellular labile iron, delayed hypoferremia, and are more susceptible to mortality from endotoxin-induced sepsis from *E*. *coli-*derived LPS [63,136]. LPS-stimulated *Lcn2*^*-/-*^ mice also exhibit elevated hallmarks of sepsis, including the expression of proinflammatory cytokines such as TNF-α and IL-18, immune cell apoptosis, and systemic organ dysfunction [63,136]. Treatment of *Lcn2*-deficient mice with the iron chelator desferrioxamine leads to protection from bacteria-free endotoxemia, suggesting an alternate role for LCN2 in the innate immune response to Gram-negative bacteria outside of its role in siderophore sequestration [136]. It is believed that LCN2 also prevents massive systemic inflammation by functioning as an anti-inflammatory agent to induce hypoferremia of infection, reduce free iron availability, and thus limit oxidative damage caused by the generation of ROS through Fenton chemistry [136].

Previous findings are mirrored by our own observations of *A*. *baumannii* bacteremia in mice, although this study appears to represent the first to use an intravenous model of infection to interrogate the role of LCN2 in Gram-negative bacteremia. Here, infected mice exhibit gene expression changes consistent with Gram-negative bacteremia and sepsis (Fig 1, S2 and S3 Tables) and *Lcn2*^*-/-*^ mice are more likely to succumb to infection (Fig 7). Given that differences in bacterial burdens were not observed in our Gram-negative bacteremia model between WT and *Lcn2*^-/-^ mice (Fig 6), we hypothesize that lethality in the latter is likely due to sepsis-related immune dysregulation, and less with the failure of the host to constrain bacterial replication through iron limitation. An important caveat to our study, however, is highlighted by the observation that different bacterial inocula can impact the outcome of infection, as exemplified by increased disease severity in the *Lcn2*^*-/-*^ mice when a higher inoculum was used (Fig 7). A probable explanation for not observing differences in burdens between WT and *Lcn2*-deficient mice in the bacteremia model is that the bacterial density simply cannot get higher than in the WT mice, for reasons including constraints on space or in resources. Indeed *in vivo* concentrations of *A*. *baumannii* above ~10^9^ CFU/mL are rarely seen for any strain during bacteremia in murine models [19,137]. In further refining our understanding of the role of LCN2 in infection, not only should inocula of varying concentrations be used, but studies should be extended beyond *A*. *baumannii* ATCC 17978 to validate hypotheses using more modern clinical isolates, as well as to additional lineages of mice. Efforts to identify the cause of mortality in the systemically infected *Lcn2*-deficient mice, heeding the aforementioned caveats, are ongoing.

Results from this study suggest that *Lcn2*-deficiency results in both bacterial replication within the lung and extrapulmonary dissemination during *A*. *baumannii* pneumonia (Fig 8). To disseminate from the lung, *A*. *baumannii* would encounter both the physical barrier of the LCN2-expressing lung epithelium, as well as LCN2-producing myeloid cells, impeding its survival and dissemination into the bloodstream. Additionally, we reason that after surviving this bottleneck, LCN2 would impair iron acquisition by the pathogen, thus hampering its proliferation in other organs. This is in line with observations during *K*. *pneumonia* lung infection, where LCN2 is required to confine bacteria to the airways and prevent systemic spread [130,131]. The replicative niche of *K*. *pneumoniae* appears to be dictated by the interactions between LCN2, transferrin, and endogenously produced siderophores, specifically LCN2-susceptible enterobactin and LCN2-resistant yersiniabactin [131]. Enterobactin promotes the replication of *K*. *pneumoniae* in the perivascular space only in the absence of LCN2, whereas yersiniabactin promotes the LCN2-independent growth of the bacteria within the airway, but is dispensable for growth in the presence of serum transferrin [131]. These observations suggest an evolutionary drive for bacteria to produce multiple siderophores that can evade various aspects of nutritional immunity, including LCN2 sequestration and the robust iron-binding capabilities of transferrin. Indeed, *A*. *baumannii* produces numerous structurally distinct siderophores including acinetobactin and pre-acinetobactin, baumannoferrins A and B, and fimsbactins A through F [85,86,110,114,138,139]. To date, only genes within the acinetobactin cluster have been shown to be essential to bacterial survival and dissemination during infection [18,19,22]. Although it is not known if any of the *A*. *baumannii* siderophores are bound by LCN2, the above results raise the intriguing possibility that multiple siderophores are produced by this pathogen to facilitate survival in host environments with differing iron availability. Indeed, data presented in this study show that LCN2 is capable of inducing iron starvation *in vitro* (Figs 4 and 5), and supports the finding that administration of recombinant LCN2 induces iron-regulated bacterial gene expression in both infected macrophages and neutropenic mice [105]. Induction of iron starvation in the presence of LCN2 is likely due to sequestration of one or more *A*. *baumannii* siderophores.

Of all the siderophores produced by *A*. *baumannii*, the baumannoferrins are least likely to be sequestered by LCN2 for two reasons. Firstly, although it was recently determined that LCN2 may be more promiscuous for binding siderophores than was previously thought, it still preferentially binds catechol and mixed type siderophores, and the baumannoferrins are hydroxamates [63,116]. Secondly, these siderophores are thought to be lipid-anchored to the cell envelope, minimizing the likelihood they are sequestered by LCN2 in the extracellular milieu [90,140]. Our data suggest that the mixed type fimsbactins siderophores may be able to evade or overwhelm LCN2 sequestration, as a mutant expressing only this siderophore biosynthetic cluster (Δ*basG bfnL*) has enhanced growth compared to WT and strains expressing acinetobactin (Δ*bfnL fbsE*) or baumannoferrins alone (Δ*basG fbsE*) (Fig 4). Notably, the Δ*basG bfnL* mutant upregulates genes in the fimsbactins cluster during iron starvation, indicating that this mutant may indeed be able to overcome LCN2-based iron limitation *in vitro*, if this translates to increased total chelator production [19]. Alternatively, Bohac *et al*. previously demonstrated that *in vitro*, fimsbactins and acinetobactin both compete for the same periplasmic binding protein, BauB [90]. Although fimsbactins form a more stable complex with iron than acinetobactin does, and both siderophores individually support the iron-dependent growth of *A*. *baumannii*, their combined presence can lead to competition and inhibition of bacterial growth. The data presented in this study indicate that in specific host niches, sequestration of acinetobactin by LCN2 may alleviate the competition between the two siderophores for BauB, and thus may facilitate iron uptake by fimsbactins. If indeed the fimsbactins siderophores are not bound by LCN2, this would further support the usage of the fimsbactins scaffold as a platform for the development of novel siderophore-antibiotic conjugates, as previously proposed [90], and would explain why some clinical isolates of *A*. *baumannii* still maintain the counterintuitive expression of this locus [86,110,141,142].

The role that LCN2 plays in inhibiting bacterial replication through iron sequestration and its broader role in the innate immune response to *A*. *baumannii* infection is still being investigated but is likely to extend beyond simply iron limitation. Several studies have highlighted the importance of neutrophils in the innate immune response to *A*. *baumannii* [43,44,143–146]. Although the exact mechanism of *A*. *baumannii* killing by neutrophils has not been elucidated, NADPH oxidase [145], ROS [147,148], myeloperoxidase [148], NETs [149,150], and β-defensins [151,152] have all been implicated as putative effectors against *A*. *baumannii* infection. Our results suggest that whilst LCN2 is unlikely to directly eliminate *A*. *baumannii* through a bactericidal mechanism, it represents another important bacteriostatic immune effector [62]. Furthermore, we observed upregulation of other neutrophil-associated nutritional immunity genes including those encoding for calprotectin subunits S100A8 and S100A9, and lactoferrin. Given that *A*. *baumannii* patients are often critically ill and that neutropenia can be a predisposing risk factor for severe disease and mortality [37,49], it is possible that neutrophil deficiency could lead to a commensurate decrease in LCN2 and other proteins that control metal distribution within the host. With the finding that LCN2 plays a critical role in infection, this raises the possibility of utilizing LCN2 as a potential therapeutic. This notion is exemplified in a study published at the time this manuscript was in preparation, demonstrating that administration of recombinant LCN2 provides partial protection from mortality of neutropenic mice infected with *A*. *baumannii* sepsis [105]. In addition to the possible supplementation of patients with recombinant LCN2 in an effort to restore the hypoferremia of infection and to sequester bacterial siderophores, LCN2 could be designed to bind additional siderophores not already accommodated by its native structure. Indeed, it has been shown that LCN2 can be structurally engineered to bind petrobactin, a stealth siderophore that is exclusively required for the virulence of *Bacillus anthracis*, and otherwise evades binding by the native protein [153,154]. This is but one possible approach to using iron acquisition in the design of new therapeutics for *A*. *baumannii* infections. It should be noted, however, that manipulating the iron balance in critically ill patients is not trivial and unforeseen negative outcomes can arise [155]. Factors confounding the use of therapeutic chelating agents can include their inadvertent use by the bacterium as an iron source, induction of iron-regulated virulence gene expression, and perturbations to host iron metabolism that lead to toxicity [156–158]. Further, recombinant proteins, such as LCN2, when administered as a therapeutic may be subject to *in vivo* proteolytic degradation, necessitating repeat administration [159]. As with the development of all novel antimicrobials, a comprehensive knowledge of the interactions between the host and pathogen is required, and in this case further highlight the need to fully understand the roles of iron, LCN2, and nutritional immunity at the *A*. *baumannii*-host interface.

As detailed above, care must be taken to observe for differences in iron homeostasis and the host innate immune response when LCN2 levels are perturbed. In infected *Lcn2*^*-/-*^ mice, a low level of LCN2 was detected by immunohistochemistry using a polyclonal goat α-mouse LCN2 antibody, despite the absence of expression in the mock-infected LCN2-deficent animals, and the known global deficiency of this protein in the knockout mice [63] (Fig 3). These results suggest that either the host, the bacteria, or both, are expressing a cross-reactive protein during infection, however the absence of immunoreactivity between the bacteria and the antibody *in vitro* indicate that this antigen is probably host-derived (S1 and S2 Figs). Although it is unknown to what epitopes the antibody binds, it is likely cross-reactive with another protein bearing a lipocalin-like fold, which consists of an eight-stranded anti-parallel β-barrel structure with an internal ligand binding site [160,161]. Lipocalins are found in vertebrates, invertebrates, plants, and some Gram-negative bacteria where in the latter they play a role in an increasingly diverse number of processes including heme-binding, the starvation response, and antimicrobial resistance [162–164]. Additionally, there are 55 genes encoding for lipocalin family proteins in the mouse genome [161]. This raises the intriguing possibility that a yet unidentified lipocalin that is induced during infection may play a role in the immune response to *A*. *baumannii*. For example, LCN2 bears ~30% identity to mouse LCN12, a poorly characterized member of the lipocalin family. Alternatively, the antibody may bind a bacterial lipocalin (Blc), where in *A*. *baumannii* this protein bears 23% identity to murine LCN2. Although immunoreactivity between bacterial proteins and the ⍺-LCN2 was not observed *in vitro* (S2 Fig), the possibility remains that the protein may be more robustly expressed and thus detected *in vivo*. Given the homology between LCN2, LCN12, and other lipocalin family proteins, one or more of the homologous proteins are likely binding to the polyclonal antibody and are targets that are also upregulated during infection. Indeed, whilst not readily explaining immunoreactivity in the infected *Lcn2*^*-/-*^ mice, a small cross-reactive protein was detected in tissues of WT infected mice (S1 Fig). The role of other members of the lipocalin protein family on the outcome of *A*. *baumannii* infection, however, is currently unknown.

Another lipocalin protein family member that may have a potential but uninterrogated role in microbial infection is LCN1, or human tear lipocalin. LCN1 is found in secretions that coat epithelial surfaces including tears, sweat, and sputum and is capable of binding a more diverse range of ligands than LCN2 such as fatty acids, phospholipids, cholesterol, and arachidonic acid [161,165–168]. In addition to lipophilic ligands, LCN1 is also capable of binding both hydroxamate and catecholate siderophores and similar to LCN2 exhibits iron-dependent antimicrobial activity [169]. Like many bacterial pathogens, the expression of multiple siderophores by *A*. *baumannii* appears at least functionally redundant [19,157]. If LCN1 functions as a broad scavenger of siderophores at epithelial surfaces, this may have contributed to the evolution of loci encoding for the production of several structurally distinct siderophores by *A*. *baumannii*. Although LCN1 concentration increases during inflammation and/or infection [170,171], the impact of this expression has not been interrogated for any pathogen and these investigations are hampered by the lack of LCN1 expression in mice [161]. Investigations into the impact of LCN1 on the iron-dependent growth of *A*. *baumannii* are ongoing.

Together the results of this study reveal that nutritional immunity, and specifically expression of LCN2, is essential to controlling the outcome of *A*. *baumannii* bacteremia and pneumonia. In addition to *Lcn2*/LCN2 being highly upregulated in the infected host, genes involved in the maintenance of the hypoferremia of infection and/or the control of other divalent metal levels saw robust expression changes during bacteremia, highlighting the potential importance of these processes to the outcome of *A*. *baumannii* infection, and providing further avenues for investigation. We have demonstrated that the lack of LCN2 expression specifically worsens the outcome of *A*. *baumannii* infections, but that the influence of this protein is likely multifactorial. Recombinant LCN2 not only inhibits the iron-dependent growth of *A*. *baumannii in vitro*, but robustly induces iron-regulated gene expression. As iron acquisition is essential to the replication of *A*. *baumannii* both *in vitro* and *in vivo*, this presents LCN2, and perhaps other lipocalins or facets of nutritional immunity, as attractive candidates for drug development against this formidable drug-resistant pathogen.

## Materials and methods

### Ethics statement

All experiments involving mice were performed in compliance with the guidelines of the National Institutes of Health, the Animal Welfare Act, the American Veterinary Medical Association, and were approved by the Vanderbilt University Institutional Animal Care and Use Committee (protocol number M1900043-00). No human participants or donors were used in this study.

### Bacterial strains and growth conditions

Experiments were conducted with *A*. *baumannii* ATCC 17978 UN and its isogenic mutants, as detailed in Table 1 [172]. *A*. *baumannii* ATCC 17978 UN differs from the alternative ATCC 17989 VU strain in that it possesses an insertion of approximately 34 different open reading frames, including genes encoding for a putative cardiolipin synthase (*cls*) and catalase. The strain used in this study was confirmed to be *A*. *baumannii* ATCC 17978 UN via polymerase chain reaction (PCR) amplifying across the *cls* gene in the UN strain using *cls-*F 5’-TCTTTCTGGCTGGTTGCTTACTCAG-3’ and *cls-*R 5’-CCGCAGCTTTCTGATTGAGACAGGC-3’, which yields a PCR product of 2347 bp in UN but not VU. Further, the identity of the strain was further confirmed using primer pairs *obgE*-UN-F 5’-GTTCAGATCCGGCCCATAT-3’ and *obgE*-R 5’-CACCACCACCAGCCATTTC-3’ which amplifies a 1128 bp in UN and *obgE*-VU-F 5’-GTTCAGATCCGGCCCATAA-3’ and *obgE-R* which amplifies the same sized fragment in VU, based on a single nucleotide polymorphism between the two strains. The results of these PCRs were further verified by whole genome sequencing. For routine cultivation *A*. *baumannii* ATCC 17978 UN was grown at 37°C in Luria-Bertani medium (LB) shaking at 180 rpm or on LB supplemented with 1.5% w/v agar (LBA).

### Murine models of *A*. *baumannii* bacteremia and pneumonia

Specific pathogen free (SPF) WT C57BL/6J mice (Stock No. 000664) and breeding pairs of LCN2-deficient mice (B6.129P2-Lcn2tm1Aade/AkiJ; stock No. 024630) were purchased from The Jackson Laboratory. LCN2-deficient mice were bred inhouse and maintained under SPF conditions prior to infection. Mice were housed with 12 h light-dark cycles and were provided food and water *ad libitum*. Experiments were performed using 6–9 week old age-matched littermate mice, and the genotypes of WT and *Lcn2*^*-/-*^ mice were confirmed by TransnetYX using PCR primers LCN2-F 5’-CAAAAGGCCTAGGTGCATCTAAGAT-3’, LCN2-R 5’-CCCTGTTCCTCCAACCCATAATAG-R’ and LCN2-reporter 5’-CCTGCCACCAAACCT-3’ for verifying excision of exons 2–5, and Neo-F 5’-GGGCGCCCGGTTCTT-3’, Neo-R 5’-CCTCGTCCTGCAGTTCATTCA-3’, and Neo-reporter 5’-ACCTGTCCGGTGCCC-3’ for verifying the presence of the replacement neomycin cassette [63].

Immunocompetent mice were infected with *A*. *baumannii* bacteremia, as previously described [19,173]. In brief, overnight cultures of WT *A*. *baumannii* were subcultured 1:100 in 30 mL of fresh LB and grown to an OD_600nm_ of approximately 2–2.5 (2–3 h). Bacteria were pelleted by centrifugation at 3,000 rpm and washed thrice with sterile phosphate buffered saline (PBS). Following the third wash, bacteria were resuspended to an OD_600nm_ of 0.35 at a dilution of 1:20 in PBS, equating to 2 x 10^9^ to 5 x 10^9^ CFU/mL. Mice were anesthetized by intraperitoneal (IP) injection with 2,2,2-tribromoethanol (Avertin) and subsequently infected by retroorbital injection with 100 μL of the prepared cells (2 x 10^8^ to 5 x 10^8^ CFU per mouse). The infection was allowed to proceed for 24 h, or during survival experiments, until mice met humane endpoint criteria. When bacterial burdens of the organs were determined, mice were euthanized and the blood, heart, lungs, liver, kidneys, and spleen were harvested. Organs were homogenized in sterile PBS using Navy Bullet Blender tubes and a Bullet Blender Tissue Homogenizer (Next Advance), serially diluted in PBS, and spot plated to LBA plates to determine the bacterial burden of each.

To determine the impact of LCN2 in multiple infection models, a murine model of pneumonia was also employed. Bacteria were prepared for infection as detailed above for bacteremia except that the cells were normalized to an OD_600nm_ of 0.35 at a dilution of 1:50, equating to approximately 7 x 10^9^ to 1 x 10^10^ CFU/mL. Mice were anesthetized with Avertin, and infected using tracheal instillation by applying 40 μL of prepared cells (2.8 x 10^8^ to 4 x 10^8^ CFU) to the nares of each mouse whereupon they inhale the inoculum. The infection was allowed to proceed for 36 h before mice were humanely euthanized, and the major organs were harvested and quantified for bacterial burdens, as described above.

### RNA extraction and analysis of host gene expression using NanoString technology

To determine changes in host gene expression during *A*. *baumannii* infection, mice were either infected systemically as described above, or mock-infected with PBS. Mice were humanely euthanized at 24 h, and organs were collected and placed in Navy Eppendorf RNA Lysis tubes (Next Advance) containing Buffer RLT (Qiagen) with 1% (v/v) β-mercaptoethanol. RNA was extracted as previously described [19], but in brief, organs were homogenized using a Bullet Blender Tissue Homogenizer (Next Advance) using cycles of 2 x 5 min at setting 8 for livers, and 2 x 5 min at setting 10 for kidneys, hearts, lungs, and spleens. Organ homogenates were transferred to fresh Bullet Blender tubes and an equal volume of phenol:chloroform:isoamyl alcohol (25:24:1) was added. Samples were homogenized for an additional 5 minutes, and then centrifuged at 15,000 rpm for 10 minutes. The upper aqueous phase of each homogenate was carefully removed and transferred to a new RNAase-free tube containing 600 μL of 70% ethanol. Samples were repeatedly inverted until a mass of nucleic acid was visible, and subsequently processed as directed using a Qiagen RNeasy Mini kit. RNA was eluted in 50 μL of nuclease-free water, quantified using a NanoDrop 8000 Spectrophotometer (Thermo Fisher), and normalized to 50–100 ng/μL. One hundred ng of extracted RNA per reaction was then hybridized with the Capture ProbeSet, and either the custom-designed Reporter CodeSet or the Myeloid Innate Immunity Reporter CodeSet for 18 h at 65°C, as directed by the manufacturer (NanoString). After hybridization, samples were processed using an nCounter FLEX analysis system (NanoString) by Vanderbilt Technologies for Advanced Genomics (VANTAGE). nSolver Analysis Software was used to process the resulting data (NanoString), where gene expression was normalized to housekeeping genes *Gapdh*, *Hprt1*, *Pgk1*, and *Tubb5* for the custom-designed CodeSet, and *Hprt1* and *Tubb5* for the Myeloid Innate Immunity panel. Background thresholding was performed by calculating the average reads detected with negative control probes in each panel, designed with no target in the sample, and delineating anything below this value as background. Gene expression is expressed as the fold-change in mice infected systemically with WT *A*. *baumannii* versus mock-infected with PBS.

### Immunohistochemistry of LCN2 in murine tissues

WT and *Lcn2*^*-/-*^ mice were infected systemically with WT *A*. *baumannii* or were mock-infected with sterile PBS, as described above. After 24 h, the mice were humanely euthanized, and intact kindeys, hearts, livers, and spleens were harvested and placed immediately in 10% neutral buffered formalin at a volume equivalent to approximately 20X the total mass of each tissue. Organs were fixed for approximately 72 h, with an exchange of formalin every 24 h. Fixed samples were transferred to tissue cassettes in formalin prior to further processing.

Following fixation, tissues were embedded in paraffin and sectioned by the Translational Pathology Shared Resource (TPSR) at Vanderbilt University Medical Center. LCN2 expression was detected using a polyclonal goat ɑ-mouse LCN2 antibody (3 μg/mL; R&D Systems; AF1857) and ɑ-goat IgG VisUCyte HRP Polymer Antibody (R&D Systems; AF1857). Immunolabeling was detected with 3,3’-diaminobenzidine (DAB) and slides were counterstained with stained with hematoxylin. Each slide was scanned, and automated digital tissue analysis of the whole slide image was performed using Aperio ImageScope Positive Pixel Count Algorithm v9. Heat maps were generated to visualize DAB staining (blue = negative, yellow = weak positive, orange = moderate positive, red = strong positive). Positivity was calculated as the number of positive pixels per total number of pixels per tissue area evaluated. The ɑ-mouse LCN2 antibody was validated, and tissues scored, by a masked pathologist. Further interpretation of cell-type associations and LCN2 localization was performed following unmasking.

### Immunoblots to assess specificity of polyclonal ɑ-LCN2 antibody

To help elucidate the possible source of the cross-reactive protein in the LCN2 immunohistochemical analysis, we performed immunoblots on organ homogenates from WT and *Lcn2*-deficient mice that were infected systemically with WT *A*. *baumannii* or mock-infected with PBS, as detailed above. After 24 h the mice were humanely euthanized, the organs were harvested and immediately deposited in ice-cold PBS containing 1% sodium dodecyl sulfate (SDS) and 1:100 protease inhibitor cocktail (Sigma-Aldrich). Organs were homogenized at 4°C, debris pelleted by centrifugation at 16,000 x g, and supernatants transferred to a new 1.5 mL tube. The protein concentration of each sample was normalized by bicinchoninic acid assay (BCA; Thermo Fisher Scientific) to 1 mg/mL, mixed with an equivalent amount of 2X SDS loading dye, and 15 μL of each sample was loaded on a 4–20% precast polyacrylamide gel (BioRad). Following polyacrylamide gel electrophoresis (PAGE) and semi-dry transfer to nitrocellulose, the membranes were briefly stained and imaged with Ponceau S to assess loading, de-stained with distilled water, and blocked with Odyssey Blocking Buffer (LI-COR) for 1 h. The primary antibody, goat α-mouse LCN2 (R&D Systems), was incubated with the membranes in blocking buffer at a concentration of 0.25 μg/mL overnight at 4°C, followed by washing thrice with PBS with Tween-20 (0.05% v/v; PBS-T). After washing, the membranes were incubated with the secondary antibody, donkey α-goat Alexa 680 (Abcam), at a dilution of 1:5000 for 2 h, and washed again thrice in PBS-T. Membranes were imaged on a ChemiDoc MP Imaging System (BioRad) using the preset settings for Alexa 680.

To further assess the selectivity of the α-LCN2 antibody we performed immunoblotting using *A*. *baumannii* WCLs. WT *A*. *baumannii* was grown in overnight in LB, and subcultured 1:1000 in triplicate into 10 mL of fresh LB with or without 200 μM of the iron chelator 2,2-dipyridyl. Bacteria were confirmed to be iron starved with 200 μM of 2,2-dipyrdyl in LB by performing growth curves using an iron-responsive reporter system, as detailed below (under “Determining the iron-regulated gene response of *A*. *baumannii* to exposure to LCN2 and 2,2-dipyridyl”). Cultures for immunoblotting were grown at 37°C with shaking at 180 rpm, and 2 mL aliquots of each culture were taken at 8, 16, and 24 h. The OD_600nm_ of each sample was recorded, bacterial cells pelleted by centrifugation, and normalized to an OD_600nm_ of 1 in PBS. A 50 μL aliquot of the normalized cells were boiled in 12.5 μL of 5X SDS-PAGE loading buffer with β-mercaptoethanol for 10 min. Gradient 4–20% SDS-PAGE gels were loaded with 15 μL of each sample. A positive control of 1 μg of recombinant LCN2 boiled in 14 μL of 1X SDS-PAGE loading buffer was prepared and 7.5 μL was loaded per gel (0.5 μg of protein). Following semi-dry transfer to nitrocellulose, blots were blocked, antibodies applied, and imaging performed, as described above. To confirm detection of *A*. *baumannii* proteins, the same samples were run with an unrelated anti-*A*. *baumannii* antibody (ɑ-HutC).

### Assessing the ability of *A*. *baumannii* to grow in the presence of recombinant LCN2

To determine if LCN2 impacts the iron-dependent growth of *A*. *baumannii in vitro*, growth curves were performed under metal limitation using bacterial strains genetically proficient for the biosynthesis of all three families of siderophores (acinetobactin and pre-acinetobactin, fimsbactins A through F, and baumannoferrins A and B), or disrupted in one or more biosynthetic pathways, as detailed in Table 1 and as previously described [19]. These strains included WT *A*. *baumannii* UN, and its isogenic Δ*basG*, Δ*bfnL*, Δ*fbsE*, Δ*basG bfnL*, Δ*basG fbsE*, Δ*bfnL fbsnE*, and Δ*basG bfnL fbsE* mutants. Bacterial strains were propagated on LBA, and single-isolated colonies were subsequently used to prepare cultures in 3 mL of LB in biological triplicate. Following overnight growth (~12 h), cultures were diluted 1:100 in fresh chelex-treated Tris Minimal Succinate media (cTMS [117]) and grown to an OD_600nm_ of approximately 1.0 (~8 h). Bacterial cells were pelleted by centrifugation at 3,000 rpm, washed thrice in sterile PBS, and resuspended at an OD_600nm_ of 0.5. Growth curves were prepared in 96-well flat-bottomed plates using cTMS media supplemented with 10% (v/v) sterile filtered human serum that was previously heat inactivated at 55°C for 30 minutes (Sigma-Aldrich; H24522). Recombinant murine LCN2 ((rmLCN2) R&D Systems; 1857-LC-050) was added to the plate in a two-fold dilution from 4 μM to 0.5 μM. To determine the iron-dependency of any growth defects, media was supplemented with 30 μM FeCl_3_, as indicated. An equivalent volume of rmLCN2 resuspension buffer of 25 mM MES and 150 mM NaCl was added as a vehicle control when rmLCN was not added. The plate was inoculated with prepared bacteria to a calculated OD_600nm_ of 0.01 (2 μL of prepared cells per well), in biological triplicate and technical quadruplicate. Growth curves were run using an Epoch2 microplate reader (BioTek), where the OD_6oonm_ was assessed every 30 minutes for 24 h. For visual clarity, data at 2 h increments are shown. Error bars represent standard error of the mean.

### Determining the iron-regulated gene response of *A*. *baumannii* to exposure to LCN2 and 2,2-dipyridyl

To determine if recombinant LCN2 and the iron chelator 2,2-dipyridyl induce iron-regulated gene expression in *A*. *baumannii*, the iron-responsive *fbsB* promoter was cloned upstream of *luxABCDE* in the plasmid p.*luxABCDE*.MU368.*tet*, generating p.P_*fbsB*_*luxABCDE*.MU368.*tet* (Table 1). In brief, primer pairs *fbsB*_*lux_*F_SacI (5’-ggacggcgcggtaccgagctcTGTGAGCACTCGTTGGAATATTAAATG-3’) and *fbsB*_*lux_*R_BamHI (5’-tcctcttgcttcatctgcaggatccAAAGCCTCCTTTGCTCAAAC-3’), designed using NEBuilder, were used to amplify the 376 bp promoter region of *fbsB* from the *A*. *baumannii* 17978 VU genome. The p.*luxABCDE*.MU368.*tet* plasmid was digested for 30 min at 37°C in CutSmart buffer using 1 unit of each HF-BamHI and HF-SacI (NEB). The reporter construct was built using Gibson Assembly following the manufacturer’s protocol (NEB), with a 7:1 molar ratio of insert:cut vector. The Gibson reaction was carried out for 1 h, prior to electroporation into *E*. *coli* DH5⍺. The construct was confirmed by sequencing prior to transformation into *A*. *baumannii* 17978 VU and was validated to be iron-responsive through exposure to the iron-chelator 2,2-dipyridyl, as well as excess iron. In brief, WT *A*. *baumannii* housing either the reporter construct, or the empty vector control were grown in biological triplicate overnight in LB supplemented with 10 μg/mL tetracycline for maintenance of the plasmids, and then subcultured 1:1000 in the same media with or without 200 μM 2,2-dipyridyl in black, clear-bottomed 96-well plates. The OD_600nm_ and luciferase activity were monitored every 30 minutes for 24 h, and the relative luminescence units were normalized to OD_600nm_ and expressed over time. Reporter assays using LCN2 were performed essentially the same, except that bacteria were grown in cTMS media supplemented with 10% human serum. Recombinant murine LCN2 was added at a concentration of 0.5 μM, which allows for growth of WT *A*. *baumannii*. Exogenous 30 μM FeCl_3_ or 0 μM LCN2 were used as comparators for gene expression under high iron and basal conditions, respectively.

## Supporting information

S1 Table Genes involved in the innate immune response to bacterial pathogens. (DOCX)

S2 Table List of highly upregulated genes in WT *A*. *baumannii* infected versus mock infected mice. (DOCX)

S3 Table Top 25 functionally enriched gene ontology terms in WT *A*. *baumannii* infected versus mock infected mice. (DOCX)

S4 Table Cell types associated with highly upregulated genes in infected mice. (DOCX)

S5 Table Localization of LCN2 immunohistochemical labeling in the kidneys of *A*. *baumannii* infected and mock infected mice. (DOCX)

S6 Table Localization of LCN2 immunohistochemical labeling in the hearts of *A*. *baumannii* infected and mock infected mice. (DOCX)

S7 Table Localization of LCN2 immunohistochemical labeling in the livers of *A*. *baumannii* infected and mock infected mice. (DOCX)

S8 Table Localization of LCN2 immunohistochemical labeling in the spleens of *A*. *baumannii* infected and mock infected mice. (DOCX)

S1 Fig LCN2 expression is detectable in *A*. *baumannii* infected WT mice by immunoblot. WT and *Lcn2*^*-/-*^ mice were infected with WT *A*. *baumannii* or mock-infected with PBS. Mice were humanely euthanized, and harvested organs were homogenized with 1% SDS and protease inhibitor cocktail. Protein concentration of each sample was normalized to 1 mg/mL prior to loading on a 4–20% SDS-PAGE gel. Following semi-dry transfer, nitrocellulose membranes were stained with Ponceau S as a loading control, de-stained, and LCN2 expression was probed with 0.25 μg/mL of goat α-mouse LCN2 polyclonal antibody followed by donkey α-goat Alexa 680 at a 1:5000 dilution. Immunoblots of the organs indicated (panels A, C, and E on the left) are shown beside their corresponding Ponceau S-stained membranes (panels B, D, and F on the right). A specific band for LCN2 is detected at ~24 kDa, primarily in the organ homogenates from infected WT mice. An unidentified non-specific band around ~12–15 kDa is apparent in the kidney homogenates of infected mice. Figures are representative of three biological repeats.
(DOCX)

S2 Fig LCN2 polyclonal antibody is not cross-reactive for bacterial proteins expressed *in vitro* under iron-deplete or replete-conditions. To further assess the selectivity of the α-LCN2 antibody, immunoblotting was performed on *A*. *baumannii* whole cell lysates, grown in LB with and without 200 μM of the iron chelator 2,2-dipyridyl. Cultures for were grown at 37°C with shaking, and aliquots of each culture were taken at 8, 16, and 24 h, as indicated. Bacterial cells were pelleted by centrifugation and then normalized to an OD_600nm_ of 1 in PBS. A 50 μL aliquot of the normalized cells was boiled in 12.5 μL of 5X SDS-PAGE loading buffer with β-mercaptoethanol for 10 min. SDS-PAGE gels were loaded with 15 μL of each sample. A positive control of 1 μg of recombinant LCN2 boiled in 14 μL of 1X SDS-PAGE loading buffer was prepared and 7.5 μL was loaded per gel (0.5 μg of protein). Following semi-dry transfer to nitrocellulose, blots were stained with Ponceau S as a loading control and then de-stained. LCN2 expression was probed with 0.20 μg/mL of goat α-mouse LCN2 polyclonal antibody followed by donkey α-goat Alexa 680 at a 1:5000 dilution (A). The blot is shown next to its corresponding Ponceau S stained-membrane, as a loading control (B). To confirm that *A*. *baumannii* proteins could be detected by immunoblotting, the same samples were also probed with an unrelated anti-*A*. *baumannii* antibody ((C) α-HutC; predicted molecular weight of 28.4 kDa).
(DOCX)

S3 Fig 2,2-dipyridyl induces iron-regulated gene expression in *A*. *baumannii*. WT *A*. *baumannii* harboring the iron-responsive p.P.*fbsB*.*luxABCDE*.*MU368*.*tet* reporter construct was grown in LB containing 10 μg/mL tetracycline, with and without the iron chelator 2,2,-dipyrdyl to confirm that the bacterial cells used in immunoblotting (S2 Fig) were iron starved. OD_600nm_ and luminescence were assessed every 30 minutes (A). For clarity, data from the timepoints utilized in immunoblotting, 8 h (B), 16 h (C) and 24 h (D) have been extracted and shown separately. Individual data points represent a single biological replicate in B, C and D. Luminescence is observed in samples treated with chelator at 8 and 16 h, indicating that an iron starvation response is induced at these timepoints. Unpaired t tests were used to assess statistical significance, where ***p <0.001 and ****p<0.0001.
(DOCX)

S4 Fig Disruption of *Lcn2* does not robustly alter the transcriptional profile in mice. WT and *Lcn2*-deficient mice were infected systemically with WT *A*. *baumannii*. At 24 h, mice were humanely euthanized, organs were harvested, and RNA was extracted. Gene expression changes in *Lcn2*-deficient versus WT mice in the kidney, heart, and liver were determined using NanoString technology and an nCounter mouse Myeloid Innate Immunology Panel. Each column represents a different gene from the panel. For clarity only genes that were downregulated ≤ -2-fold or upregulated ≥ +2 in at least one organ are shown.
(DOCX)

S5 Fig Disruption of fimsbactins biosynthesis may impair growth in the presence of LCN2. WT *A*. *baumannii* (A) and its isogenic siderophore mutants defective in the production of acinetobactin and pre-acinetobactin (B), baumannoferrins A and B (C), or fimsbactins (D) were grown in cTMS media supplemented with LCN2 at the concentrations indicated. Growth was assessed by determining the optical density at OD_600nm_ over 24 h. Data are representative of two experiments performed in biological triplicate.
(DOCX)
