# Supplementary material for: Lipocalin-2 is an essential component of the innate immune response to Acinetobacter baumannii infection
Source: PLoS Pathog. 2022 Sep 2;18(9):e1010809. doi: 10.1371/journal.ppat.1010809 (PMC9477428; doi:10.1371/journal.ppat.1010809)
Supplement: S1 Table — (DOCX) [file ppat.1010809.s001.docx]

**S1 Table. Genes involved in the innate immune response to bacterial pathogens.**

| **Gene name** | **Protein name; description** | **Function** | **Reference** |
| --- | --- | --- | --- |
| ***Cp*** | Ceruloplasmin | Binds serum copper, loads transferrin with iron. | [1] |
| ***Bdh2*** | 3-Hydroxybutyrate dehydrogenase 2 | Facilitates the production of 2,5-dihydroxybenzoic acid (2,5-DHBA), a mammalian siderophore that is structurally similar to enterobactin and binds LCN2. | [2,3] |
| ***Hamp*** | Hepcidin | Binds to and internalizes ferroportin, sequestering iron within macrophages and hepatocytes. Decreases intestinal absorption of iron. | [4] |
| ***Lcn2*** | Lipocalin-2 | Sequesters catechol siderophores, including the mammalian siderophore 2,5-DHBA. Binding of 2,5-DHBA reduces the availability of free intracellular iron. | [2,5–8] |
| ***Ltf*** | Lactoferrin | Iron-binding glycoprotein responsible for sequestering the metal at mucosal surfaces. | [9–12] |
| ***Slc40a1*** | Solute carrier family 40 member 1; ferroportin | Only known iron exporter, transports iron from the cytosol. Required for iron homeostasis. Expression is regulated by hepcidin. Also exports zinc, cobalt, nickel, and manganese. | [13–19] |
| ***Slc11a2*** | Solute carrier family 11 member 2; divalent metal transporter 1 ((DMT1) also known as natural-resistance associated macrophage protein 2 (NRAMP2)) | Promotes uptake of dietary iron from the duodenal lumen by enterocytes. Facilitates transport of other divalent metals including manganese, cobalt, copper, zinc, nickel, lead, and cadmium. | [20,21] |
| ***S100a8*** | S100 calcium-binding protein A8; subunit of calprotectin | As a heterodimer with S100A9 it sequesters transition metals including iron, manganese, and zinc to inhibit bacterial growth. | [22,23] |
| ***S100a9*** | S100 calcium-binding protein A9; subunit of calprotectin | As a heterodimer with S100A8 it sequesters transition metals including iron, manganese, and zinc to inhibit bacterial growth. | [22,23] |
| ***Slc30a10*** | Solute carrier family 30 member 10; ZNT10 | Exports manganese from enterocytes and hepatocytes. Contributes to manganese homeostasis and alleviates manganese toxicity. | [24–26] |
| ***Slc39a14*** | Solute carrier family 39 member 14; ZIP14 | Involved in manganese homeostasis. Promotes uptake of manganese, and subsequent excretion, from the liver and pancreas. Facilitates cellular uptake of divalent metals such as iron and zinc. | [27–29] |

**S1 Table. Genes involved in the innate immune response to bacterial pathogens (cont’d).**

| **Gene name** | **Protein name; description** | **Function** | **References** |
| --- | --- | --- | --- |
| ***Slc39a8*** | Solute carrier family 39 member 8; ZIP8 | Cellular uptake of divalent metals including zinc, iron, manganese, and cadmium from the extracellular space into the cytoplasm. | [30–32] |
| ***Slc11a1*** | Solute carrier family 11 member 1; NRAMP1 | Transports divalent metals, primarily iron and manganese, out of the phagosome. Provides resistance to intracellular pathogens. | [33–37] |
| ***Mt1*** | Metallothionein 1 | Small cysteine-rich protein involved in heavy metal storage, detoxification, and transport. Can coordinate up to seven molecules of zinc or cadmium. | [38,39] |
| ***Mt2*** | Metallothionein 2 | Small cysteine-rich protein involved in heavy metal storage, detoxification, and transport. Can coordinate up to seven molecules of zinc or cadmium. | [39,40] |
| ***Slc30a2*** | Solute carrier family 30 member 2; ZNT2 | Zinc transporter that may protect cells from cellular toxicity by facilitating zinc import into vesicular compartments, secretes zinc from mammary glands. | [41,42] |
| ***Slc30a5*** | Solute carrier family 30 member 5; ZNT5 | May transport zinc into secretory granules of pancreatic beta cells, involved in activation of alkaline phosphatases. | [43,44] |
| ***Slc30a1*** | Solute carrier family 30 member 1; ZNT1 | Exports cellular zinc into extracellular space. Attenuates growth of intracellular pathogens. | [45] |
| ***Slc39a11*** | Solute carrier family 39 member 11; ZIP11 | Cellular zinc importer. Facilitates zinc uptake from the stomach and colon. | [46,47] |
| ***Slc39a4*** | Solute carrier family 39 member 4; ZIP4 | Required for zinc homeostasis. Localized to apical surface of enterocytes and facilitates uptake of dietary zinc. | [48,49] |
| ***Slc39a7*** | Solute carrier family 39 member 7; ZIP7 | Transports zinc from the Golgi apparatus and endoplasmic reticulum into the cell. | [50,51] |
| ***Slc30a4*** | Solute carrier family 30 member 4; ZNT4 | Provides zinc to zinc-dependent proteins in the Golgi apparatus. Required for zinc export in mammary cells, deficiency results in lethal milk mouse. | [52,53] |
| ***Slc30a7*** | Solute carrier family 30 member 7; ZNT7 | Transports cytoplasmic zinc into the Golgi apparatus for zinc storage. Involved in activation of alkaline phosphatases. | [43,54] |
| ***Flvcr1b*** | Feline leukemia virus subgroup C receptor 1 | Exports heme from mitochondria into cytosol. | [55] |
| ***Hba1*** | Alpha hemoglobin; subunit of hemoglobin | Subunit of hemoglobin, the iron-containing, oxygen-transporting component of red blood cells. | [56] |
| ***Hmox1*** | Heme oxygenase 1 | Catalyzes the degradation of heme to release biliverdin, free ferrous iron, and carbon dioxide. Inducible and thought to confer resistance to oxidative stress. | [57,58] |

**S1 Table. Genes involved in the innate immune response to bacterial pathogens (cont’d).**

| **Gene name** | **Protein name; description** | **Function** | **References** |
| --- | --- | --- | --- |
| ***Cybb*** | Cytochrome B-245 beta chain; subunit of Cytochrome B-245 and NADPH oxidase | Together with cytochrome B-245 alpha chain forms the main catalytic subunit of NADPH oxidase (Nox2), which is the major microbicidal oxidase of phagocytes. | [59,60] |
| ***Mpo*** | Myeloperoxidase | Peroxidase enzyme that produces hypohalous acids which kill bacteria and other pathogens. Released upon neutrophil degranulation. | [61] |
| ***Nos2*** | Nitric oxide synthase 2 | Enzyme catalyzing the conversion of L-arginine to nitric oxide, a reactive free radical. NOS2 is inducible and in mice contributes to the defense against intracellular pathogens. | [62–64] |
| ***Sod1*** | Superoxide dismutase 1; [Cu-Zn]-SOD | Copper and zinc requiring enzyme that catalyzes the conversion of superoxide radicals to hydrogen peroxide and oxygen. Localized to cytosol. Involved in protection against oxidative stress. | [65] |
| ***Sod2*** | Superoxide dismutase 2; [Mn]-SOD | Manganese requiring enzyme that catalyzes the conversion of superoxide radicals to hydrogen peroxide and oxygen. Localized to mitochondria. Involved in protection against oxidative stress. | [65] |
| ***S100a7*** | Psoriasin | Secreted by skin epithelial cells, sequesters zinc, activates neutrophils, and exhibits antibacterial activity. | [66–68] |
| ***Il22*** | Interleukin 22 | Maintains barrier function, promotes recruitment of immune cells, promotes defense mechanisms. | [69] |
| ***Gapdh*** | Glyceraldehyde-3-phosphate dehydrogenase | Housekeeping. Enzyme catalyzing the conversion of glyceraldehyde-3-phosphate to D-glycerate 1,3-bisphosphate | [70] |
| ***Hprt1*** | Hypoxanthine Phosphoribosyltransferase 1. | Housekeeping. Enzyme that catalyzes the conversion of hypoxanthine to inosine monophosphate and guanine to guanosine monophosphate. | [70] |
| ***Pgk1*** | Phosphoglycerate kinase 1 | Housekeeping. Enzyme that catalyzes the conversion of 1,3-diphosphoglycerate to 3-phosphoglycerate. | [70] |
| ***Tubb5*** | Tubulin beta-5 chain | Housekeeping. Major component of microtubules. | [70] |

**References**

1. Holmberg CG, Laurell C-B, Gjertsen P. Investigations in serum copper. II. Isolation of the copper containing protein, and a description of some of its properties. Acta Chemica Scandinavica. 1948;2: 550–556. doi:10.3891/acta.chem.scand.02-0550

2. Liu Z, Reba S, Chen WD, Porwal SK, Boom WH, Petersen RB, et al. Regulation of mammalian siderophore 2,5-DHBA in the innate immune response to infection. Journal of Experimental Medicine. 2014;211: 1197–1213. doi:10.1084/jem.20132629

3. Devireddy LR, Hart DO, Goetz DH, Green MR. A mammalian siderophore synthesized by an enzyme with a bacterial homolog involved in enterobactin production. Cell. 2010;141: 1006–1017. doi:10.1016/j.cell.2010.04.040

4. Ganz T. Hepcidin, a key regulator of iron metabolism and mediator of anemia of inflammation. Blood. 2003;102: 783–788. doi:10.1182/blood-2003-03-0672

5. Flo TH, Smith KD, Sato S, Rodriguez DJ, Holmes MA, Strong RK, et al. Lipocalin 2 mediates an innate immune response to bacterial infection by sequestrating iron. Nature. 2004;432: 917–921. doi:10.1038/nature03104

6. Goetz DH, Holmes MA, Borregaard N, Bluhm ME, Raymond KN, Strong RK. The neutrophil lipocalin NGAL is a bacteriostatic agent that interferes with siderophore-mediated iron acquisition. Mol Cell. 2002;10: 1033–43. doi:10.1016/s1097-2765(02)00708-6

7. Holmes MA, Paulsene W, Jide X, Ratledge C, Strong RK. Siderocalin (Lcn 2) also binds carboxymycobactins, potentially defending against mycobacterial infections through iron sequestration. Structure. 2005;13: 29–41. doi:10.1016/j.str.2004.10.009

8. Song E, Ramos S v., Huang X, Liu Y, Botta A, Sung HK, et al. Holo-lipocalin-2–derived siderophores increase mitochondrial ROS and impair oxidative phosphorylation in rat cardiomyocytes. Proc Natl Acad Sci U S A. 2018;115: 1576–1581. doi:10.1073/pnas.1720570115

9. Levay P, Viljoen M. Lactoferrin: a general review. Haematologica. 1995;80: 252–267. doi:10.3324/%x

10. Lönnerdal B, Iyer S. Lactoferrin: Molecular structure and biological function. Annual Review of Nutrition. 1995;15: 93–110. doi:10.1146/annurev.nu.15.070195.000521

11. Johanson B, Virtanen AI, Tweit RC, Dodson RM. Isolation of an iron-containing red protein from human milk. Acta Chemica Scandinavica. 1960;14: 510–512. doi:10.3891/acta.chem.scand.14-0510

12. Groves ML. The isolation of a red protein from milk. J Am Chem Soc. 1960;82: 3345–3350. doi:10.1021/ja01498a029

13. Drakesmith H, Nemeth E, Ganz T. Ironing out ferroportin. Cell Metabolism. 2015;22: 777–787. doi:10.1016/j.cmet.2015.09.006

14. Donovan A, Brownlie A, Zhou Y, Shepard J, Pratt SJ, Moynihan J, et al. Positional cloning of zebrafish ferroportin1 identifies a conserved vertebrate iron exporter. Nature. 2000;403: 776–781. doi:10.1038/35001596

15. Madejczyk MS, Ballatori N. The iron transporter ferroportin can also function as a manganese exporter. Biochimica et Biophysica Acta - Biomembranes. 2012;1818: 651–657. doi:10.1016/j.bbamem.2011.12.002

16. Donovan A, Lima CA, Pinkus JL, Pinkus GS, Zon LI, Robine S, et al. The iron exporter ferroportin/Slc40a1 is essential for iron homeostasis. Cell Metabolism. 2005;1: 191–200. doi:10.1016/j.cmet.2005.01.003

17. Deshpande CN, Ruwe TA, Shawki A, Xin V, Vieth KR, Valore E v., et al. Calcium is an essential cofactor for metal efflux by the ferroportin transporter family. Nature Communications. 2018;9: 3075. doi:10.1038/s41467-018-05446-4

18. Mitchell CJ, Shawki A, Ganz T, Nemeth E, Mackenzie B. Functional properties of human ferroportin, a cellular iron exporter reactive also with cobalt and zinc. American Journal of Physiology - Cell Physiology. 2014;306: C450. doi:10.1152/ajpcell.00348.2013

19. Nemeth E, Tuttle MS, Powelson J, Vaughn MD, Donovan A, Ward DMV, et al. Hepcidin regulates cellular iron efflux by binding to ferroportin and inducing its internalization. Science (1979). 2004;306: 2090–2093. doi:10.1126/science.1104742

20. Andrews NC. The iron transporter DMT1. International Journal of Biochemistry and Cell Biology. 1999;31: 991–994. doi:10.1016/S1357-2725(99)00065-5

21. Garrick MD, Dolan KG, Horbinski C, Ghio AJ, Higgins D, Porubcin M, et al. DMT1: A mammalian transporter for multiple metals. BioMetals. 2003;16: 41–54. doi:10.1023/A:1020702213099

22. Corbin BD, Seeley EH, Raab A, Feldmann J, Miller MR, Torres VJ, et al. Metal chelation and inhibition of bacterial growth in tissue abscesses. Science (1979). 2008;319: 962–965. doi:10.1126/science.1152449

23. Zygiel EM, Nolan EM. Transition metal sequestration by the host-defense protein calprotectin. Annual Review of Biochemistry. 2018;87: 621–643. doi:10.1146/annurev-biochem-062917-012312

24. Quadri M, Federico A, Zhao T, Breedveld GJ, Battisti C, Delnooz C, et al. Mutations in SLC30A10 cause parkinsonism and dystonia with hypermanganesemia, polycythemia, and chronic liver disease. American Journal of Human Genetics. 2012;90: 467–477. doi:10.1016/j.ajhg.2012.01.017

25. Mercadante CJ, Prajapati M, Conboy HL, Dash ME, Herrera C, Pettiglio MA, et al. Manganese transporter Slc30a10 controls physiological manganese excretion and toxicity. Journal of Clinical Investigation. 2019;129: 5442–5461. doi:10.1172/JCI129710

26. Leyva-Illades D, Chen P, Zogzas CE, Hutchens S, Mercado JM, Swaim CD, et al. SLC30A10 is a cell surface-localized manganese efflux transporter, and parkinsonism-causing mutations block its intracellular trafficking and efflux activity. Journal of Neuroscience. 2014;34: 14079–14095. doi:10.1523/JNEUROSCI.2329-14.2014

27. Jenkitkasemwong S, Akinyode A, Paulus E, Weiskirchen R, Hojyo S, Fukada T, et al. SLC39A14 deficiency alters manganese homeostasis and excretion resulting in brain manganese accumulation and motor deficits in mice. Proc Natl Acad Sci U S A. 2018;115: E1769–E1778. doi:10.1073/pnas.1720739115

28. Liuzzi JP, Aydemir F, Nam H, Knutson MD, Cousins RJ. Zip14 (Slc39a14) mediates non-transferrin-bound iron uptake into cells. Proc Natl Acad Sci U S A. 2006;103: 13612–13617. doi:10.1073/pnas.0606424103

29. Pinilla-Tenas JJ, Sparkman BK, Shawki A, Illing AC, Mitchell CJ, Zhao N, et al. Zip14 is a complex broad-scope metal-ion transporter whose functional properties support roles in the cellular uptake of zinc and nontransferrin-bound iron. American Journal of Physiology - Cell Physiology. 2011;301: C862-71. doi:10.1152/ajpcell.00479.2010

30. Jenkitkasemwong S, Wang CY, MacKenzie B, Knutson MD. Physiologic implications of metal-ion transport by ZIP14 and ZIP8. BioMetals. 2012;25: 643–655. doi:10.1007/s10534-012-9526-x

31. He L, Girijashanker K, Dalton TP, Reed J, Li H, Soleimani M, et al. ZIP8, member of the solute-carrier-39 (SLC39) metal-transporter family: Characterization of transporter properties. Molecular Pharmacology. 2006;70: 171–180. doi:10.1124/mol.106.024521

32. Wang CY, Jenkitkasemwong S, Duarte S, Sparkman BK, Shawki A, Mackenzie B, et al. ZIP8 is an iron and zinc transporter whose cell-surface expression is up-regulated by cellular iron loading. Journal of Biological Chemistry. 2012;287: 34032–34043. doi:10.1074/jbc.M112.367284

33. Wessling-Resnick M. Nramp1 and other transporters involved in metal withholding during infection. Journal of Biological Chemistry. 2015;290: 18984–18990. doi:10.1074/jbc.R115.643973

34. Gruenheid S, Pinner E, Desjardins M, Gros P. Natural resistance to infection with intracellular pathogens: The Nramp1 protein is recruited to the membrane of the phagosome. Journal of Experimental Medicine. 1997;185: 717–730. doi:10.1084/jem.185.4.717

35. Jabado N, Jankowski A, Dougaparsad S, Picard V, Grinstein S, Gros P. Natural resistance to intracellular infections: Natural resistance-associated macrophage protein 1 (NRAMP1) functions as a pH-dependent manganese transporter at the phagosomal membrane. Journal of Experimental Medicine. 2000;192: 1237–1247. doi:10.1084/jem.192.9.1237

36. Cellier MF, Courville P, Campion C. Nramp1 phagocyte intracellular metal withdrawal defense. Microbes and Infection. 2007;9: 1662–1670. doi:10.1016/j.micinf.2007.09.006

37. Sheldon JR, Skaar EP. Metals as phagocyte antimicrobial effectors. Current Opinion in Immunology. 2019;60: 1–9. doi:10.1016/j.coi.2019.04.002

38. Suhy DA, Simon KD, Linzer DIH, O’Halloran T v. Metallothionein is part of a zinc-scavenging mechanism for cell survival under conditions of extreme zinc deprivation. Journal of Biological Chemistry. 1999;274: 9183–9192. doi:10.1074/jbc.274.14.9183

39. Rahman MT, Karim MM. Metallothionein: A potential link in the regulation of zinc in nutritional immunity. Biological Trace Element Research. 2018;182: 1–13. doi:10.1007/s12011-017-1061-8

40. Drozd A, Wojewska D, Peris-Díaz MD, Jakimowicz P, Krȩzel A. Crosstalk of the structural and zinc buffering properties of mammalian metallothionein-2. Metallomics. 2018;10: 595–613. doi:10.1039/c7mt00332c

41. Lopez V, Kelleher SL. Zinc transporter-2 (ZnT2) variants are localized to distinct subcellular compartments and functionally transport zinc. Biochemical Journal. 2009;422: 43–52. doi:10.1042/BJ20081189

42. Palmiter RD, Cole TB, Findley SD. ZnT-2, a mammalian protein that confers resistance to zinc by facilitating vesicular sequestration. EMBO Journal. 1996;15: 1784–1791. doi:10.1002/j.1460-2075.1996.tb00527.x

43. Suzuki T, Ishihara K, Migaki H, Matsuura W, Kohda A, Okumura K, et al. Zinc transporters, ZnT5 and ZnT7, are required for the activation of alkaline phosphatases, zinc-requiring enzymes that are glycosylphosphatidylinositol-anchored to the cytoplasmic membrane. Journal of Biological Chemistry. 2005;280: 637–643. doi:10.1074/jbc.M411247200

44. Kambe T, Narita H, Yamaguchi-Iwai Y, Hirose J, Amano T, Sugiura N, et al. Cloning and characterization of a novel mammalian zinc transporter, zinc transporter 5, abundantly expressed in pancreatic β cells. Journal of Biological Chemistry. 2002;277: 19049–19055. doi:10.1074/jbc.M200910200

45. Nishito Y, Kambe T. Zinc transporter 1 (ZNT1) expression on the cell surface is elaborately controlled by cellular zinc levels. Journal of Biological Chemistry. 2019;294: 15686–15697. doi:10.1074/jbc.RA119.010227

46. Cousins RJ, Martin AB, Aydemir TB, Guthrie GJ, Samuelson DA, Chang SM. Gastric and colonic zinc transporter ZIP11 (Slc39a11) in mice responds to dietary zinc and exhibits nuclear localization. Journal of Nutrition. 2013;143: 1882–1888. doi:10.3945/jn.113.184457

47. Yu Y, Wu A, Zhang Z, Yan G, Zhang F, Zhang L, et al. Characterization of the GufA subfamily member SLC39A11/Zip11 as a zinc transporter. Journal of Nutritional Biochemistry. 2013;24: 1697–1708. doi:10.1016/j.jnutbio.2013.02.010

48. Dufner-Beattie J, Wang F, Kuo YM, Gitschier J, Eide D, Andrews GK. The acrodermatitis enteropathica gene ZIP4 encodes a tissue-specific, zinc-regulated zinc transporter in mice. Journal of Biological Chemistry. 2003;278: 33474–33481. doi:10.1074/jbc.M305000200

49. Andrews GK. Regulation and function of *Zip4*, the acrodermatitis enteropathica gene. Biochemical Society Transactions. 2008;36: 1242–1246. doi:10.1042/BST0361242

50. Woodruff G, Bouwkamp CG, de Vrij FM, Lovenberg T, Bonaventure P, Kushner SA, et al. The zinc transporter SLC39A7 (ZIP7) is essential for regulation of cytosolic zinc levels. Molecular Pharmacology. 2018;94: 1092–1100. doi:10.1124/mol.118.112557

51. Huang L, Kirschke CP, Zhang Y, Yan YY. The *ZIP7* gene (*Slc39a7*) encodes a zinc transporter involved in zinc homeostasis of the Golgi apparatus. Journal of Biological Chemistry. 2005;280: 15456–15463. doi:10.1074/jbc.M412188200

52. Huang L, Gitschier J. A novel gene involved in zinc transport is deficient in the lethal milk mouse. Nature Genetics. 1997;17: 292–297. doi:10.1038/ng1197-292

53. McCormick NH, Kelleher SL. ZnT4 provides zinc to zinc-dependent proteins in the trans-Golgi network critical for cell function and Zn export in mammary epithelial cells. American Journal of Physiology - Cell Physiology. 2012;303: C291-7. doi:10.1152/ajpcell.00443.2011

54. Kirschke CP, Huang L. ZnT7, a novel mammalian zinc transporter, accumulates zinc in the Golgi apparatus. Journal of Biological Chemistry. 2003;278: 4096–4102. doi:10.1074/jbc.M207644200

55. Chiabrando D, Marro S, Mercurio S, Giorgi C, Petrillo S, Vinchi F, et al. The mitochondrial heme exporter FLVCR1b mediates erythroid differentiation. Journal of Clinical Investigation. 2012;122: 4569–4579. doi:10.1172/JCI62422

56. Baldwin JM. Structure and function of haemoglobin. Progress in Biophysics and Molecular Biology. 1976;29: 225–320. doi:10.1016/0079-6107(76)90024-9

57. Maines MD, Trakshel GM, Kutty RK. Characterization of two constitutive forms of rat liver microsomal heme oxygenase. Only one molecular species of the enzyme is inducible. Journal of Biological Chemistry. 1986;261: 411–419. doi:10.1016/S0021-9258(17)42488-4

58. Morse D, Choi AMK. Heme oxygenase-1: The “emerging molecule” has arrived. American Journal of Respiratory Cell and Molecular Biology. 2002;27: 8–16. doi:10.1165/ajrcmb.27.1.4862

59. Rada B, Hably C, Meczner A, Timár C, Lakatos G, Enyedi P, et al. Role of Nox2 in elimination of microorganisms. Seminars in Immunopathology. 2008;30: 237–253. doi:10.1007/s00281-008-0126-3

60. Cross AR, Segal AW. The NADPH oxidase of professional phagocytes - Prototype of the NOX electron transport chain systems. Biochimica et Biophysica Acta - Bioenergetics. 2004;1657: 1–22. doi:10.1016/j.bbabio.2004.03.008

61. Klebanoff SJ. Myeloperoxidase: friend and foe. Journal of Leukocyte Biology. 2005;77: 598–625. doi:10.1189/jlb.1204697

62. Chakravortty D, Hensel M. Inducible nitric oxide synthase and control of intracellular bacterial pathogens. Microbes and Infection. 2003;5: 621–627. doi:10.1016/S1286-4579(03)00096-0

63. Geller DA, Lowenstein CJ, Shapiro RA, Nussler AK, di Silvio M, Wang SC, et al. Molecular cloning and expression of inducible nitric oxide synthase from human hepatocytes. Proc Natl Acad Sci U S A. 1993;90: 3491–3495. doi:10.1073/pnas.90.8.3491

64. Bogdan C. Nitric oxide and the immune response. Nature Immunology. 2001;2: 907–916. doi:10.1038/ni1001-907

65. Zelko IN, Mariani TJ, Folz RJ. Superoxide dismutase multigene family: A comparison of the CuZn-SOD (SOD1), Mn-SOD (SOD2), and EC-SOD (SOD3) gene structures, evolution, and expression. Free Radical Biology and Medicine. 2002;33: 337–349. doi:10.1016/S0891-5849(02)00905-X

66. Brodersen DE, Nyborg J, Kjeldgaard M. Zinc-binding site of an S100 protein revealed. Two crystal structures of Ca^2+^-bound human psoriasin (S100A7) in the Zn^2+^-loaded and Zn^2+^-free states. Biochemistry. 1999;38: 1695–1704. doi:10.1021/bi982483d

67. Gläser R, Harder J, Lange H, Bartels J, Christophers E, Schröder JM. Antimicrobial psoriasin (S100A7) protects human skin from *Escherichia coli* infection. Nature Immunology. 2005;6: 57–64. doi:10.1038/ni1142

68. Zheng Y, Niyonsaba F, Ushio H, Ikeda S, Nagaoka I, Okumura K, et al. Microbicidal protein psoriasin is a multifunctional modulator of neutrophil activation. Immunology. 2008;124: 357–367. doi:10.1111/j.1365-2567.2007.02782.x

69. Dudakov JA, Hanash AM, van den Brink MRM. Interleukin-22: Immunobiology and pathology. Annual Review of Immunology. 2015;33: 747–785. doi:10.1146/annurev-immunol-032414-112123

70. NanoString. nCounter® Technology | NanoString Technologies. [cited 11 Nov 2019]. Available: https://www.nanostring.com/scientific-content/technology-overview/ncounter-technology
