# Supplementary material for: Lipocalin-2 is an essential component of the innate immune response to Acinetobacter baumannii infection
Source: PLoS Pathog. 2022 Sep 2;18(9):e1010809. doi: 10.1371/journal.ppat.1010809 (PMC9477428; doi:10.1371/journal.ppat.1010809)
Supplement: S2 Table — (DOCX) [file ppat.1010809.s002.docx]

**S2 Table. List of highly upregulated genes in WT *A. baumannii* infected versus mock infected mice.**

| **Gene name^a^** | **Protein name; description** | **Function** | **References** |
| --- | --- | --- | --- |
| ***Acod1*** | Cis-aconitate decarboxylase (ACOD1) | Catalyzes the production of itaconate, produced in response to pathogen infection, mediates oxidative stress and pathogen processing. | [1] |
| ***Adamts4*** | A disintegrin and metalloproteinase with thrombospondin (ADAMTS) motifs 4 (ADAMTS4) | Major proteinase responsible for the degradation of proteoglycans, remodels extracellular matrix (ECM) following infection and can contribute to lung pathology. | [2] |
| ***Ccl2*** | Chemokine (C-C motif) ligand 2 (CCL2; also known as monocyte chemoattractant protein 1 (MCP1) and small inducible cytokine A2) | Recruits monocytes and macrophages to the site of inflammation. | [3] |
| ***Ccl5*** | Chemokine (C-C motif) ligand 5 (CCL5) | Proinflammatory cytokine that recruits immune cells during inflammation/infection including T cells, dendritic cells, eosinophils, mast cells, and basophils. | [4] |
| ***Cd274*** | Cluster of differentiation 274 (CD274; also known as programmed death-ligand 1 (PD-L1) or B7 homolog 1 (B7-H1)) | Suppresses the adaptive arm of the immune system, may exacerbate acute infections such as sepsis. | [5] |
| ***Cdkn1a*** | Cyclin-dependent kinase inhibitor 1A (CDKN1A; also known as p21  p21^Cip1^, p21^Waf1^ or CDK-interacting protein 1) | Multifaceted/purpose regulator. Cell cycle inhibitor with roles in inducing senescence, apoptosis, cell migration, and differentiation, amongst others. | [6] |
| ***Cebpd*** | CCAAT/enhancer-binding protein delta (CEBPD) | Transcription factor that regulates diverse biological processes including cell differentiation, migration, metabolism and the immune response. Activated by inflammatory factors including TNF-α, NF-Kβ, and LPS. | [7] |
| ***Csf3*** | Colony-stimulating factor 3(CSF3; also known as granulocyte colony-stimulating factor (G-CSF or GCSF)) | Impacts the survival, proliferation, and differentiation of neutrophils. Induced during infection upon exposure to inflammatory mediators such as TNF-α, IL-1 and LPS. Can be used to help alleviate neutropenia. | [8] |
| ***Cxcl1*** | Chemokine (C-X-C motif) ligand 1 (CXCL1) | Acts as a chemoattractant primarily for neutrophils. Recruits and activates neutrophils to facilitate killing of microbes but promoting NADPH oxidase–mediated ROS production and NET formation. | [9,10] |
| ***Cxcl10*** | Chemokine (C-X-C motif) ligand 10 (CXCL10; also known as interferon gamma-induced protein 10 (IP-10) or small-inducible cytokine B10) | Chemoattractant for various immune cells including macrophages, T cells, dendritic cells, and NK cells. Elevated during inflammatory conditions including infectious diseases. | [11] |

**S2 Table. List of highly upregulated genes in WT *A. baumannii* infected versus mock infected mice (cont’d).**

| **Gene name^a^** | **Protein name; description** | **Function** | **References** |
| --- | --- | --- | --- |
| ***Cxcl2*** | Chemokine (C-X-C motif) ligand 2 (CXCL2, also known as growth regulated oncogene-2 (GRO2), GRO protein beta (GROβ) or macrophage inflammatory protein 2-alpha (MIP2ɑ) | Secreted by monocytes, macrophages, and mast cells. Attracts neutrophils to the site of tissue inflammation. May play a role in airway remodeling by inducing airway smooth muscle cell migration. | [12,13] |
| ***Cxcl3*** | Chemokine (C-X-C motif) ligand 3 (CXCL3; also known GRO3, GRO𝛾, or MIP2β) | Controls migration and adhesion of monocytes. May play a role in airway remodeling by inducing airway smooth muscle cell migration. | [13,14] |
| ***Cxcl5*** | Chemokine (C-X-C motif) ligand 5 (CXCL5; also known as epithelial-derived neutrophil-activating peptide 78 (ENA-78)) | Produced following stimulation of cells with TNF-α and IL-1. Promotes activation and migration of neutrophils. | [15] |
| ***Cxcl9*** | Chemokine (C-X-C motif) ligand 9 (CXCL9; also known as monokine induced by gamma interferon (MIG)) | Regulates the migration, activation, and differentiation of immune cells. Recruits NK cells, cytotoxic lymphocytes, and macrophages. Induced by IFN-𝛾. | [16] |
| ***Il1rn*** | Interleukin-1 receptor antagonist (IL1RN) | Plays a role in host defense against endotoxic injury. Inhibits the biological function of IL-1. | [17] |
| ***Il6*** | Interleukin-6 (IL6) | Functions as a pro-inflammatory cytokine and is produced by macrophages upon exposure to pathogen-associated molecular patterns (PAMPs). Induces hepcidin production, which facilitates destruction of ferroportin and reduces serum iron. Also induces expression of the divalent metal importer ZIP14 on hepatocytes, leading to their internalization. | [18] |
| ***Irf7*** | Interferon regulatory factory 7 (IRF7) | Transcription factor regulating the expression of interferon (IFN) genes. Expression is triggered in response to pathogenic infection. Functions as the master regulator of type I IFN production. IFNs function by inducing expression of genes involved in many processes, including immune modulation. | [19] |
| ***Myc*** | c-MYC | Transcription factor that activates expression of growth-promoting genes or represses those involved in cell growth arrest and adhesion. | [20] |
| ***Plaur*** | Urokinase plasminogen activator surface receptor (uPAR; also known as cluster of differentiation 87 (CD87) or urokinase receptor) | Part of the plasminogen activation system involved in tissue reorganization. Essential to the activation of plasminogen to plasmin, which facilitates the degradation of the ECM via the proteolytic cascade. | [21,22] |

**S2 Table. List of highly upregulated genes in WT *A. baumannii* infected versus mock infected mice (cont’d).**

| **Gene name^a^** | **Protein name; description** | **Function** | **References** |
| --- | --- | --- | --- |
| ***S100a8*** | S100 calcium-binding protein A8; subunit of calprotectin | As a heterodimer with S100A9 it sequesters transition metals including iron, manganese, and zinc to inhibit bacterial growth. | [23,24] |
| ***S100a9*** | S100 calcium-binding protein A9; subunit of calprotectin | As a heterodimer with S100A8 it sequesters transition metals including iron, manganese, and zinc to inhibit bacterial growth. | [23,24] |
| ***Serpine1*** | Plasminogen activator inhibitor-1 (PAI-1; also known as serpin E1 or endothelial plasminogen activator inhibitor) | Serine protease inhibitor that inhibits the activity of urokinase plasminogen activator, preventing the formation of plasmin. Stabilizes the formation of thrombus, promoting wound healing. | [25] |
| ***Socs3*** | Suppressor of cytokine signaling 3 (SOCS3) | Suppresses cytokine activity by inhibiting  Janus kinase–signal transducer and activator of transcription (JAK-STAT) signaling. Expression can be stimulated by pathogens. | [26] |
| ***Sphk1*** | Sphingosine kinase 1 (SPHK1) | Participates in sphingolipid metabolism, functioning to phosphorylate sphingosine to sphingosine-1 phosphate, a lipid signaling molecule that regulates cell proliferation and survival. | [27] |
| ***Tnfaip3*** | Tumor necrosis factor, alpha-induced protein 3 (TNFAIP3; also known as or A20) | Induced by TNF and functions to suppress NF-Kβ activation. Suppresses endotoxin and NF-Kβ mediated inflammation. Mutations lead to spontaneous inflammation and may contribute to autoimmunity. | [28] |

**^a^**Genes in this list were upregulated ≥ 12-fold in each the kidney, heart, and spleen. No genes were downregulated to a comparable amount in all three organs.

**References**

1. Wu R, Chen F, Wang N, Tang D, Kang R. ACOD1 in immunometabolism and disease. Cellular and Molecular Immunology. 2020;17: 822–833. doi:10.1038/s41423-020-0489-5

2. Boyd DF, Sanders CJ, Bajracharya R, Diercks AH, Thomas PG. ADAMTS4 modulates lung tissue repair following lethal influenza A infection in mice. The Journal of Immunology. 2016;196: 78.22.

3. Deshmane SL, Kremlev S, Amini S, Sawaya BE. Monocyte chemoattractant protein-1 (MCP-1): An overview. Journal of Interferon and Cytokine Research. 2009;29: 313–325. doi:10.1089/jir.2008.0027

4. Marques RE, Guabiraba R, Russo RC, Teixeira MM. Targeting CCL5 in inflammation. Expert Opinion on Therapeutic Targets. 2013;17: 1439–1460. doi:10.1517/14728222.2013.837886

5. Brown KE, Freeman GJ, Wherry EJ, Sharpe AH. Role of PD-1 in regulating acute infections. Current Opinion in Immunology. 2010;22: 397–401. doi:10.1016/j.coi.2010.03.007

6. Kreis NN, Louwen F, Yuan J. The multifaceted p21 (Cip1/Waf1/CDKN1A) in cell differentiation, migration and cancer therapy. Cancers (Basel). 2019;11: 1220. doi:10.3390/cancers11091220

7. Ko CY, Chang WC, Wang JM. Biological roles of CCAAT/enhancer-binding protein delta during inflammation. Journal of Biomedical Science. 2015;22: 6. doi:10.1186/s12929-014-0110-2

8. Roberts AW. G-CSF: A key regulator of neutrophil production, but that’s not all! Growth Factors. 2005;23: 33–41. doi:10.1080/08977190500055836

9. Jin L, Batra S, Douda DN, Palaniyar N, Jeyaseelan S. CXCL1 contributes to host defense in polymicrobial sepsis via modulating T cell and neutrophil functions. The Journal of Immunology. 2014;193: 3549–3558. doi:10.4049/jimmunol.1401138

10. Sawant K v., Poluri KM, Dutta AK, Sepuru KM, Troshkina A, Garofalo RP, et al. Chemokine CXCL1 mediated neutrophil recruitment: Role of glycosaminoglycan interactions. Scientific Reports. 2016;6: 1–8. doi:10.1038/srep33123

11. Liu M, Guo S, Hibbert JM, Jain V, Singh N, Wilson NO, et al. CXCL10/IP-10 in infectious diseases pathogenesis and potential therapeutic implications. Cytokine and Growth Factor Reviews. 2011;22: 121–130. doi:10.1016/j.cytogfr.2011.06.001

12. de Filippo K, Dudeck A, Hasenberg M, Nye E, van Rooijen N, Hartmann K, et al. Mast cell and macrophage chemokines CXCL1/CXCL2 control the early stage of neutrophil recruitment during tissue inflammation. Blood. 2013;121: 4930–4937. doi:10.1182/blood-2013-02-486217

13. Al-Alwan LA, Chang Y, Mogas A, Halayko AJ, Baglole CJ, Martin JG, et al. Differential roles of CXCL2 and CXCL3 and their receptors in regulating normal and asthmatic airway smooth muscle cell migration. The Journal of Immunology. 2013;191: 2731–2741. doi:10.4049/jimmunol.1203421

14. Olson TS, Ley K. Chemokines and chemokine receptors in leukocyte trafficking. American Journal of Physiology-Regulatory, Integrative and Comparative Physiology. 2002;283: R7–R28. doi:10.1152/ajpregu.00738.2001

15. Chang MS, McNinch J, Basu R, Simonet S. Cloning and characterization of the human neutrophil-activating peptide (ENA-78) gene. Journal of Biological Chemistry. 1994;269: 25277–25282. doi:10.1016/s0021-9258(18)47243-2

16. Tokunaga R, Zhang W, Naseem M, Puccini A, Berger MD, Soni S, et al. CXCL9, CXCL10, CXCL11/CXCR3 axis for immune activation – A target for novel cancer therapy. Cancer Treatment Reviews. 2018;63: 40–47. doi:10.1016/j.ctrv.2017.11.007

17. Arena WP, Malyak M, Guthridge CJ, Gabay C. Interleukin-1 receptor antagonist: Role in biology. Annual Review of Immunology. 1998;16: 27–55. doi:10.1146/annurev.immunol.16.1.27

18. Tanaka T, Narazaki M, Kishimoto T. Il-6 in inflammation, immunity, and disease. Cold Spring Harbor Perspectives in Biology. 2014;6: a016295. doi:10.1101/cshperspect.a016295

19. Ning S, Pagano JS, Barber GN. IRF7: Activation, regulation, modification and function. Genes and Immunity. 2011;12: 399–414. doi:10.1038/gene.2011.21

20. Dang C v., O’Donnell KA, Zeller KI, Nguyen T, Osthus RC, Li F. The c-Myc target gene network. Seminars in Cancer Biology. 2006;16: 253–264. doi:10.1016/j.semcancer.2006.07.014

21. Plesner T, Behrendt N, Ploug M. Structure, function and expression on blood and bone marrow cells of the urokinase‐type plasminogen activator receptor, uPAR. Stem Cells. 1997;15: 398–408. doi:10.1002/stem.150398

22. Mahmood N, Mihalcioiu C, Rabbani SA. Multifaceted role of the urokinase-type plasminogen activator (uPA) and its receptor (uPAR): Diagnostic, prognostic, and therapeutic applications. Frontiers in Oncology. 2018;8: 24. doi:10.3389/fonc.2018.00024

23. Corbin BD, Seeley EH, Raab A, Feldmann J, Miller MR, Torres VJ, et al. Metal chelation and inhibition of bacterial growth in tissue abscesses. Science (1979). 2008;319: 962–965. doi:10.1126/science.1152449

24. Zygiel EM, Nolan EM. Transition metal sequestration by the host-defense protein calprotectin. Annual Review of Biochemistry. 2018;87: 621–643. doi:10.1146/annurev-biochem-062917-012312

25. Iwaki T, Urano T, Umemura K. PAI-1, progress in understanding the clinical problem and its aetiology. British Journal of Haematology. 2012;157: 291–298. doi:10.1111/j.1365-2141.2012.09074.x

26. Carow B, Rottenberg ME. SOCS3, a major regulator of infection and inflammation. Frontiers in Immunology. 2014;5: 58. doi:10.3389/fimmu.2014.00058

27. Maceyka M, Payne SG, Milstien S, Spiegel S. Sphingosine kinase, sphingosine-1-phosphate, and apoptosis. Biochimica et Biophysica Acta - Molecular and Cell Biology of Lipids. 2002;1585: 193–201. doi:10.1016/S1388-1981(02)00341-4

28. Das T, Chen Z, Hendriks RW, Kool M. A20/tumor necrosis factor α-induced protein 3 in immune cells controls development of autoinflammation and autoimmunity: Lessons from mouse models. Frontiers in Immunology. 2018;9: 104. doi:10.3389/fimmu.2018.00104
