# Supplementary material for: Lipocalin-2 is an essential component of the innate immune response to Acinetobacter baumannii infection
Source: PLoS Pathog. 2022 Sep 2;18(9):e1010809. doi: 10.1371/journal.ppat.1010809 (PMC9477428; doi:10.1371/journal.ppat.1010809)
Supplement: S3 Table — (DOCX) [file ppat.1010809.s003.docx]

**S3 Table. Functionally enriched gene ontology terms in WT *A. baumannii* infected versus mock infected mice.**

| **Gene ontology (GO) code** | **Enriched GO term** | **Count^a^** | **^b^Percent represented** | ***p*-value** | **Benjamini adjusted *p*-value** [1] |
| --- | --- | --- | --- | --- | --- |
| GO:0034097 | Response to cytokine | 18 | 72 | 1.33 x 10^-19^ | 2.45 x 10^-16^ |
| GO:0071345 | Cellular response to cytokine stimulus | 17 | 68 | 2.30 x 10^-19^ | 4.90 x 10^-16^ |
| GO:0006954 | Inflammatory response | 17 | 68 | 1.64 x 10^-18^ | 3.49 x 10^-15^ |
| GO:0006952 | Defense response | 20 | 80 | 6.74 x 10^-17^ | 2.40 x 10^-13^ |
| GO:0032103 | Positive regulation of response to external stimuli | 13 | 52 | 2.45 x 10^-16^ | 4.72 x 10^-13^ |
| GO:0032496 | Response to lipopolysaccharide | 14 | 56 | 5.56 x 10^-16^ | 1.18 x 10^-12^ |
| GO:0002237 | Response to molecule of bacterial origin | 14 | 56 | 8.68 x 10^-16^ | 1.89 x 10^-12^ |
| GO:0019221 | Cytokine-mediated signaling pathway | 13 | 52 | 2.36 x 10^-15^ | 4.95 x 10^-12^ |
| GO:0002684 | Positive regulation of immune system process | 16 | 64 | 3.20 x 10^-15^ | 6.84 x 10^-12^ |
| GO:0033993 | Response to lipid | 17 | 68 | 3.34 x 10^-15^ | 7.08 x 10^-12^ |
| GO:0048584 | Positive regulation of response to stimulus | 20 | 80 | 3.70 x 10^-15^ | 7.78 x 10^-12^ |
| GO:0009617 | Response to bacterium | 15 | 60 | 1.33 x 10^-14^ | 2.83 x 10^-11^ |
| GO:0030595 | Leukocyte chemotaxis | 11 | 44 | 3.36 x 10^-14^ | 7.12 x 10^-11^ |
| GO:0032101 | Regulation of response to external stimulus | 15 | 60 | 8.47 x 10^-14^ | 1.80 x 10^-10^ |
| GO:0006955 | Immune response | 17 | 68 | 1.01 x 10^-13^ | 2.14 x 10^-10^ |
| GO:0002682 | Regulation of immune process | 17 | 68 | 1.23 x 10^-13^ | 2.61 x 10^-10^ |
| GO:0002690 | Positive regulation of leukocyte chemotaxis | 9 | 36 | 1.69 x 10^-13^ | 3.95 x 10^-10^ |
| GO:0009607 | Response to biotic stimulus | 16 | 64 | 3.80 x 10^-13^ | 8.08 x 10^-10^ |
| GO:0060326 | Cell chemotaxis | 11 | 44 | 5.96 x 10^-13^ | 1.27 x 10^-9^ |
| GO:0002688 | Regulation of leukocyte chemotaxis | 9 | 36 | 9.83 x 10^-13^ | 2.09 x 10^-9^ |
| GO:0010033 | Response to organic substance | 21 | 84 | 1.27 x 10^-12^ | 2.71 x 10^-9^ |
| GO:0070098 | Chemokine-mediated signaling pathway | 8 | 32 | 1.38 x 10^-12^ | 2.95 x 10^-9^ |
| GO:0070887 | Cellular response to chemical stimulus | 20 | 80 | 2.40 x 10^-12^ | 5.11 x 10^-9^ |
| GO:0050900 | Leukocyte migration | 11 | 44 | 2.63 x 10^-12^ | 5.60 x 10^-9^ |
| GO:0030335 | Positive regulation of cell migration | 12 | 48 | 3.77 x 10^-12^ | 8.00 x 10^-9^ |

^a^Total number of genes out of 25 encompassed by the GO term.

^b^Percentage of genes encompassed by the GO term.

**References**

1. Benjamini Y, Hochberg Y. Controlling the false discovery rate: A practical and powerful approach to multiple testing. Journal of the Royal Statistical Society: Series B (Methodological). 1995;57: 289–300. doi:10.1111/j.2517-6161.1995.tb02031.x
