# Supplementary material for: Lipocalin-2 is an essential component of the innate immune response to Acinetobacter baumannii infection
Source: PLoS Pathog. 2022 Sep 2;18(9):e1010809. doi: 10.1371/journal.ppat.1010809 (PMC9477428; doi:10.1371/journal.ppat.1010809)
Supplement: S4 Table — (DOCX) [file ppat.1010809.s004.docx]

**S4** **Table. Cell types associated with highly upregulated genes in infected mice.**

| **Cell type^a^** | **# of genes represented in NanoString panel** | **# of genes represented in upregulated group** | **Percentage**  **of total genes** |
| --- | --- | --- | --- |
| **Neutrophil** | 137 | 11 | 8.03 |
| **Eosinophil** | 103 | 3 | 2.91 |
| **Mast cell** | 147 | 7 | 4.76 |
| **Dendritic cell** | 264 | 10 | 3.79 |
| **Monocytes-macrophages** | 332 | 14 | 4.22 |

^a^Cell type associations were determined using the NanoString nCounter Mouse Myeloid Innate Immunity Panel gene to cell type association analysis [1].

**References**

1. nCounter® Myeloid Innate Immunity Panel |NanoString Technologies. [cited 9 Nov 2021]. Available:https://www.nanostring.com/products/ncounter-assays-panels/immunology/ myeloid-innate-immunity/
