# Supplementary material for: Lipocalin-2 is an essential component of the innate immune response to Acinetobacter baumannii infection
Source: PLoS Pathog. 2022 Sep 2;18(9):e1010809. doi: 10.1371/journal.ppat.1010809 (PMC9477428; doi:10.1371/journal.ppat.1010809)
Supplement: S6 Table — (DOCX) [file ppat.1010809.s006.docx]

**S6 Table. Localization of LCN2 immunohistochemical labeling in the hearts of *A. baumannii* infected and mock infected mice.**

| **Mouse-treatment** | **Localization within heart^a^** | | |
| --- | --- | --- | --- |
|  | **Cardiomyocytes** | **Interstitium** | **Vasculature** |
| WT-mock | - | + | - |
| WT-infected | + | ++ | - |
| *Lcn2^-/-^* -mock | - | - | - |
| *Lcn2^-/-^* -infected | - | + | - |

^a^+ represents low expression, ++ moderate expression, - no detectable expression.
