# Supplementary material for: Lipocalin-2 is an essential component of the innate immune response to Acinetobacter baumannii infection
Source: PLoS Pathog. 2022 Sep 2;18(9):e1010809. doi: 10.1371/journal.ppat.1010809 (PMC9477428; doi:10.1371/journal.ppat.1010809)
Supplement: S7 Table — (DOCX) [file ppat.1010809.s007.docx]

**S7 Table. Localization of LCN2 immunohistochemical labeling in the livers of *A. baumannii* infected and mock infected mice.**

| **Mouse-treatment** | **Localization within liver^a^** | | | |
| --- | --- | --- | --- | --- |
|  | **Hepatocytes** | **Kupfer cells** | **Bile ducts** | **Vasculature** |
| WT-mock | ++ | +++ | - | - |
| WT-infected | ++++ | ++++ | - | - |
| *Lcn2^-/-^* -mock | - | - | - | - |
| *Lcn2^-/-^* -infected | + | ++ | - | - |

^a^+ represents low expression, ++ moderate expression, +++ high expression, ++++ very high expression, - no detectable expression.
