# Supplementary material for: Lipocalin-2 is an essential component of the innate immune response to Acinetobacter baumannii infection
Source: PLoS Pathog. 2022 Sep 2;18(9):e1010809. doi: 10.1371/journal.ppat.1010809 (PMC9477428; doi:10.1371/journal.ppat.1010809)
Supplement: S8 Table — (DOCX) [file ppat.1010809.s008.docx]

**S8 Table. Localization of LCN2 immunohistochemical labeling in the spleens of *A. baumannii* infected and mock infected mice.**

| **Mouse-treatment** | **Localization within spleen^a^** | |
| --- | --- | --- |
|  | **Red pulp** | **White pulp** |
| WT-mock | ++ | - |
| WT-infected | +++ | + |
| *Lcn2^-/-^* -mock | - | - |
| *Lcn2^-/-^* -infected | ++ | + |

^a^+ represents low expression, ++ moderate expression, +++ high expression, - no detectable expression.
