# Supplementary material for: Lipocalin-2 is an essential component of the innate immune response to Acinetobacter baumannii infection
Source: PLoS Pathog. 2022 Sep 2;18(9):e1010809. doi: 10.1371/journal.ppat.1010809 (PMC9477428; doi:10.1371/journal.ppat.1010809)
Supplement: S1 Fig — WT and Lcn2-/- mice were infected with WT A. baumannii or mock-infected with PBS. Mice were humanely euthanized, and harvested organs were homogenized with 1% SDS and protease inhibitor cocktail. Protein concentration of each sample was normalized to 1 mg/mL prior to loading on a 4–20% SDS-PAGE gel. Following semi-dry transfer, nitrocellulose membranes were stained with Ponceau S as a loading control, de-stained, and LCN2 expression was probed with 0.25 μg/mL of goat α-mouse LCN2 polyclonal antibody followed by donkey α-goat Alexa 680 at a 1:5000 dilution. Immunoblots of the organs indicated (panels A, C, and E on the left) are shown beside their corresponding Ponceau S-stained membranes (panels B, D, and F on the right). A specific band for LCN2 is detected at ~24 kDa, primarily in the organ homogenates from infected WT mice. An unidentified non-specific band around ~12–15 kDa is apparent in the kidney homogenates of infected mice. Figures are representative of three biological repeats. (DOCX) [file ppat.1010809.s009.docx]

**S1 Figure. LCN2 expression is detectable in *A. baumannii* infected WT mice by immunoblot.** WT and *Lcn2^-/-^* mice were infected with WT *A. baumannii* or mock-infected with PBS. Mice were humanely euthanized, and harvested organs were homogenized with 1% SDS and protease inhibitor cocktail. Protein concentration of each sample was normalized to 1 mg/mL prior to loading on a 4-20% SDS-PAGE gel. Following semi-dry transfer, nitrocellulose membranes were stained with Ponceau S as a loading control, de-stained, and LCN2 expression was probed with 0.25 µg/mL of goat α-mouse LCN2 polyclonal antibody followed by donkey α-goat Alexa 680 at a 1:5000 dilution. Immunoblots of the organs indicated (panels A, C, and E on the left) are shown beside their corresponding Ponceau S-stained membranes (panels B, D, and F on the right). A specific band for LCN2 is detected at ~24 kDa, primarily in the organ homogenates from infected WT mice. An unidentified non-specific band around ~12-15 kDa is apparent in the kidney homogenates of infected mice. Figures are representative of three biological repeats.
