# Supplementary material for: Lipocalin-2 is an essential component of the innate immune response to Acinetobacter baumannii infection
Source: PLoS Pathog. 2022 Sep 2;18(9):e1010809. doi: 10.1371/journal.ppat.1010809 (PMC9477428; doi:10.1371/journal.ppat.1010809)
Supplement: S2 Fig — To further assess the selectivity of the α-LCN2 antibody, immunoblotting was performed on A. baumannii whole cell lysates, grown in LB with and without 200 μM of the iron chelator 2,2-dipyridyl. Cultures for were grown at 37°C with shaking, and aliquots of each culture were taken at 8, 16, and 24 h, as indicated. Bacterial cells were pelleted by centrifugation and then normalized to an OD600nm of 1 in PBS. A 50 μL aliquot of the normalized cells was boiled in 12.5 μL of 5X SDS-PAGE loading buffer with β-mercaptoethanol for 10 min. SDS-PAGE gels were loaded with 15 μL of each sample. A positive control of 1 μg of recombinant LCN2 boiled in 14 μL of 1X SDS-PAGE loading buffer was prepared and 7.5 μL was loaded per gel (0.5 μg of protein). Following semi-dry transfer to nitrocellulose, blots were stained with Ponceau S as a loading control and then de-stained. LCN2 expression was probed with 0.20 μg/mL of goat α-mouse LCN2 polyclonal antibody followed by donkey α-goat Alexa 680 at a 1:5000 dilution (A). The blot is shown next to its corresponding Ponceau S stained-membrane, as a loading control (B). To confirm that A. baumannii proteins could be detected by immunoblotting, the same samples were also probed with an unrelated anti-A. baumannii antibody ((C) α-HutC; predicted molecular weight of 28.4 kDa). (DOCX) [file ppat.1010809.s010.docx]

**S2 Figure. LCN2 polyclonal antibody is not cross-reactive for bacterial proteins expressed *in vitro* under iron-deplete or replete-conditions.** To further assess the selectivity of the α-LCN2 antibody, immunoblotting was performed on *A. baumannii* whole cell lysates, grown in LB with and without 200 µM of the iron chelator 2,2-dipyridyl. Cultures for were grown at 37 °C with shaking, and aliquots of each culture were taken at 8, 16, and 24 h, as indicated. Bacterial cells were pelleted by centrifugation and then normalized to an OD_600nm_ of 1 in PBS. A 50 µL aliquot of the normalized cells was boiled in 12.5 µL of 5X SDS-PAGE loading buffer with β-mercaptoethanol for 10 min. SDS-PAGE gels were loaded with 15 µL of each sample. A positive control of 1 µg of recombinant LCN2 boiled in 14 µL of 1X SDS-PAGE loading buffer was prepared and 7.5 µL was loaded per gel (0.5 µg of protein). Following semi-dry transfer to nitrocellulose, blots were stained with Ponceau S as a loading control and then de-stained. LCN2 expression was probed with 0.20 µg/mL of goat α-mouse LCN2 polyclonal antibody followed by donkey α-goat Alexa 680 at a 1:5000 dilution (A). The blot is shown next to its corresponding Ponceau S-stained membrane, as a loading control (B). To confirm that *A. baumannii* proteins could be detected by immunoblotting, the same samples were also probed with an unrelated anti-*A. baumannii* antibody ((C) α-HutC; predicted molecular weight of 28.4 kDa).
