# Supplementary material for: Lipocalin-2 is an essential component of the innate immune response to Acinetobacter baumannii infection
Source: PLoS Pathog. 2022 Sep 2;18(9):e1010809. doi: 10.1371/journal.ppat.1010809 (PMC9477428; doi:10.1371/journal.ppat.1010809)
Supplement: S3 Fig — WT A. baumannii harboring the iron-responsive p.P.fbsB.luxABCDE.MU368.tet reporter construct was grown in LB containing 10 μg/mL tetracycline, with and without the iron chelator 2,2,-dipyrdyl to confirm that the bacterial cells used in immunoblotting (S2 Fig) were iron starved. OD600nm and luminescence were assessed every 30 minutes (A). For clarity, data from the timepoints utilized in immunoblotting, 8 h (B), 16 h (C) and 24 h (D) have been extracted and shown separately. Individual data points represent a single biological replicate in B, C and D. Luminescence is observed in samples treated with chelator at 8 and 16 h, indicating that an iron starvation response is induced at these timepoints. Unpaired t tests were used to assess statistical significance, where ***p <0.001 and ****p<0.0001. (DOCX) [file ppat.1010809.s011.docx]

**S3 Figure. 2,2-dipyridyl induces iron-regulated gene expression in *A. baumannii*.** WT *A. baumannii* harboring the iron-responsive p.P.*fbsB.luxABCDE.MU368.tet* reporter construct was grown in LB containing 10 µg/mL tetracycline, with and without the iron chelator 2,2,-dipyrdyl to confirm that the bacterial cells used in immunoblotting (S2 Fig) were iron starved. OD_600nm_ and luminescence were assessed every 30 minutes (A). For clarity, data from the timepoints utilized in immunoblotting, 8 h (B), 16 h (C) and 24 h (D) have been extracted and shown separately. Individual data points represent a single biological replicate in B, C and D. Luminescence is observed in samples treated with chelator at 8 and 16 h, indicating that an iron starvation response is induced at these timepoints. Unpaired t tests were used to assess statistical significance, where ***p <0.001 and ****p<0.0001.
