# Supplementary material for: Lipocalin-2 is an essential component of the innate immune response to Acinetobacter baumannii infection
Source: PLoS Pathog. 2022 Sep 2;18(9):e1010809. doi: 10.1371/journal.ppat.1010809 (PMC9477428; doi:10.1371/journal.ppat.1010809)
Supplement: S4 Fig — WT and Lcn2-deficient mice were infected systemically with WT A. baumannii. At 24 h, mice were humanely euthanized, organs were harvested, and RNA was extracted. Gene expression changes in Lcn2-deficient versus WT mice in the kidney, heart, and liver were determined using NanoString technology and an nCounter mouse Myeloid Innate Immunology Panel. Each column represents a different gene from the panel. For clarity only genes that were downregulated ≤ -2-fold or upregulated ≥ +2 in at least one organ are shown. (DOCX) [file ppat.1010809.s012.docx]

**S4 Figure. Disruption of *Lcn2* does not robustly alter the transcriptional profile in mice.** WT and *Lcn2*­-deficient mice were infected systemically with WT *A. baumannii*. At 24 h, mice were humanely euthanized, organs were harvested, and RNA was extracted. Gene expression changes in *Lcn2*-deficient versus WT mice in the kidney, heart, and liver were determined using NanoString technology and an nCounter mouse Myeloid Innate Immunology Panel. Each column represents a different gene from the panel. For clarity only genes that were downregulated ≤ -2-fold or upregulated ≥ +2 in at least one organ are shown.
