# Supplementary material for: Lipocalin-2 is an essential component of the innate immune response to Acinetobacter baumannii infection
Source: PLoS Pathog. 2022 Sep 2;18(9):e1010809. doi: 10.1371/journal.ppat.1010809 (PMC9477428; doi:10.1371/journal.ppat.1010809)
Supplement: S5 Fig — WT A. baumannii (A) and its isogenic siderophore mutants defective in the production of acinetobactin and pre-acinetobactin (B), baumannoferrins A and B (C), or fimsbactins (D) were grown in cTMS media supplemented with LCN2 at the concentrations indicated. Growth was assessed by determining the optical density at OD600nm over 24 h. Data are representative of two experiments performed in biological triplicate. (DOCX) [file ppat.1010809.s013.docx]

**S5 Figure. Disruption of fimsbactins biosynthesis may impair growth in the presence of LCN2.** WT *A. baumannii* (A) and its isogenic siderophore mutants defective in the production of acinetobactin and pre-acinetobactin (B), baumannoferrins A and B (C), or fimsbactins (D) were grown in cTMS media supplemented with LCN2 at the concentrations indicated. Growth was assessed by determining the optical density at OD_600nm_ over 24 h. Data are representative of two experiments performed in biological triplicate.
